# Unusual Fluorescence Behavior of Pyrene-Amine Containing Dendrimers

**DOI:** 10.3390/molecules24224083

**Published:** 2019-11-12

**Authors:** Andrea Ruiu, Mireille Vonlanthen, Sandra M. Rojas-Montoya, Israel González-Méndez, Ernesto Rivera

**Affiliations:** Instituto de Investigaciones en Materiales, Universidad Nacional Autónoma de México, Circuito Exterior Ciudad Universitaria, C.P. 04510 Ciudad de México, Mexico; mireille.vonlanthen@gmail.com (M.V.); sandrismrm@hotmail.com (S.M.R.-M.); israelgonzalezme@gmail.com (I.G.-M.)

**Keywords:** pyrene, cyclen, dendrimers, fluorescence, quenching

## Abstract

A new class of pyrene-based dendrimers, characterized by the presence of a 1,4,7,10-Tetraazacyclododecane (cyclen) unit as the core, was studied by SSF (steady-state fluorescence) and SPC (single-photon counting fluorescence). The photophysical behavior of these dendrimers was studied in THF, DMF and DMSO solution. The typical signals for pyrene-labeled molecules were recorded in each solvent, showing the representative fluorescence spectra: the corresponding emissions of monomer and excimer of the pyrene chromophore are observed. Unexpectedly, the typical quenching of tertiary amine on the pyrene emission was not observed in these dendrimers. Quenching studies were performed by adding up to 3 equivalents of trifluoroacetic acid (TFA). To our knowledge, this is the first report of pyrene’s unquenching behavior by a tertiary amine.

## 1. Introduction

Dendrimers are regular structures with a spherical tridimensional arrangement, which confers it interesting physical and chemical properties. The first reports date back from the 1980s, and since then, dendritic molecules have been studied in depth [1,2,3]. Photoactive units are often incorporated into the dendritic structures in order to obtain luminescent systems. The chromophores can be incorporated either in the periphery, branches or core. The luminescent units can interact with an environmental entity in order to form an excimer, exciplex or a quenched system, thereby allowing to study their behavior [4,5,6,7,8]. This kind of construct has found applications in material research for photovoltaic and light-emitting devices, as well as in medical research for drug delivery and sensors.

Pyrene is one of the most studied chromophores due to its impressive photochemical properties: high quantum yield, well defined absorption and emission bands, long life time and the ability to form excimers are just a representative list of the properties which allow an extensive study of pyrene-labeled molecules [9,10,11,12,13]. In fact, this molecule has been used in various chemical structures, such as peptides [13,14], oligothiophenes [15], molecules exhibiting energy transfer [16,17,18], and metal organic frameworks [19,20].

Different models are used to investigate the intermolecular phenomena of pyrene-containing systems, correlated to a fluorescence time scale. The most used are the Birks’ scheme, the Fluorescence Blob Model and the Model Free Analysis (MFA). This last method has proved to be very efficient to study the dendrimeric structures in detail [21,22].

As a photoactive unit, pyrene fluorescence is sensitive to different kinds of quencher such as oxygen, heavy atoms, amine, and nitro-groups, among others [23,24,25,26,27,28,29,30,31]. This behavior allows the system to be used as a sensor of these quenchers. The quenching can happen by different pathways depending on the type of quencher. The most common are the intersystem crossing, the electron exchange and the photoinduced electron transfer. Usually, the amino group quenching is reversible by the protonation of the nitrogen atom and its quenching power increases from primary to tertiary amines [32,33,34].

In this work, we report the optical and photophysical characterization of three generations of Frechet-type dendrimers bearing cyclen as core and pyrene units in the periphery and two model compounds. All the investigated constructs were studied by SSF and SPF and the obtained results were analyzed by MFA. Furthermore, quenching studies of the amine-pyrene systems were done for model compounds and dendrimers.

## 2. Results and Discussion 

### 2.1. Analysis of the Model Compounds and the Pyrene Dendrimers

#### 2.1.1. Model Compounds

**Figure 1:**
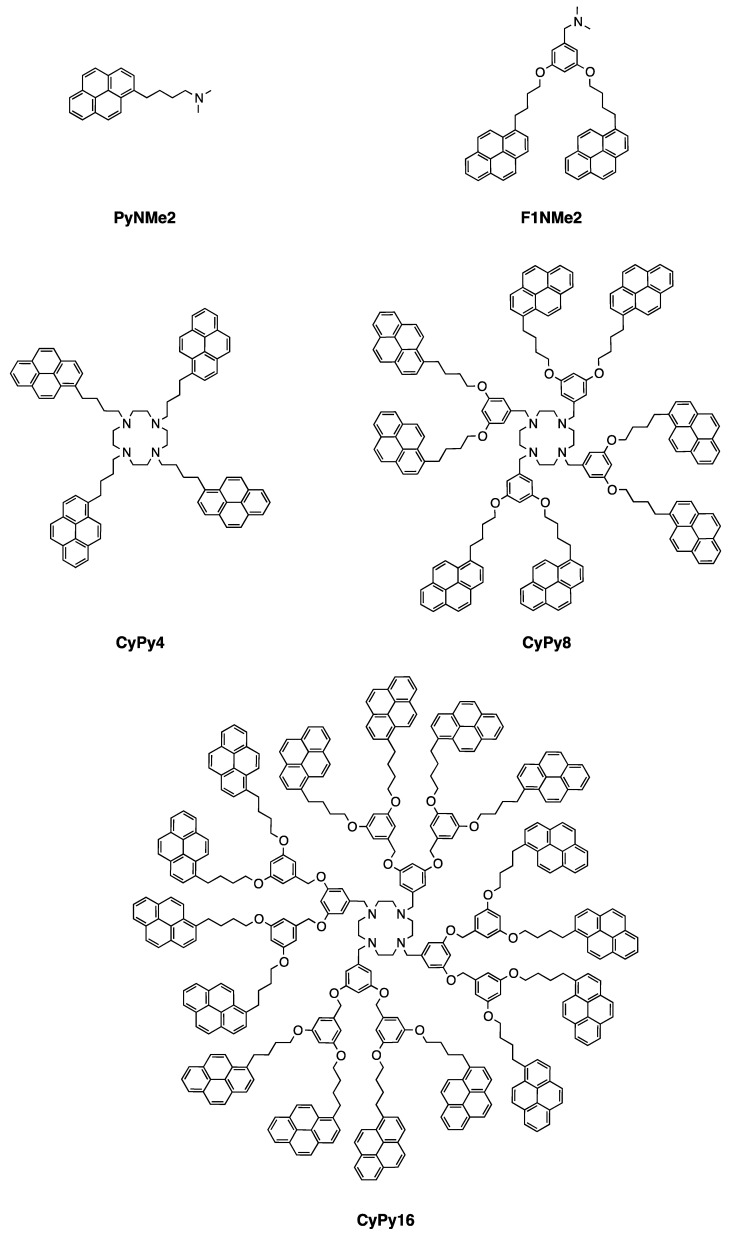
Structures of pyrene-based model compounds and dendrimers.

In order to study the photophysical properties of the dendritic systems, model compounds (Figure 1) were synthesized and their fluorescence spectra were analyzed. Their synthesis is reported in the Supporting Information. These reference compounds **PyNMe2** and **F1NMe2** represent a portion of **CyPy4** and **CyPy8** dendrimers, and were studied in order to idealize and simplify the studies of pyrene–amine interactions. SSF and SPC analysis has been performed in tetrahydrofuran (THF), a typical solvent for pyrene-labeled systems.

**Figure 2:**
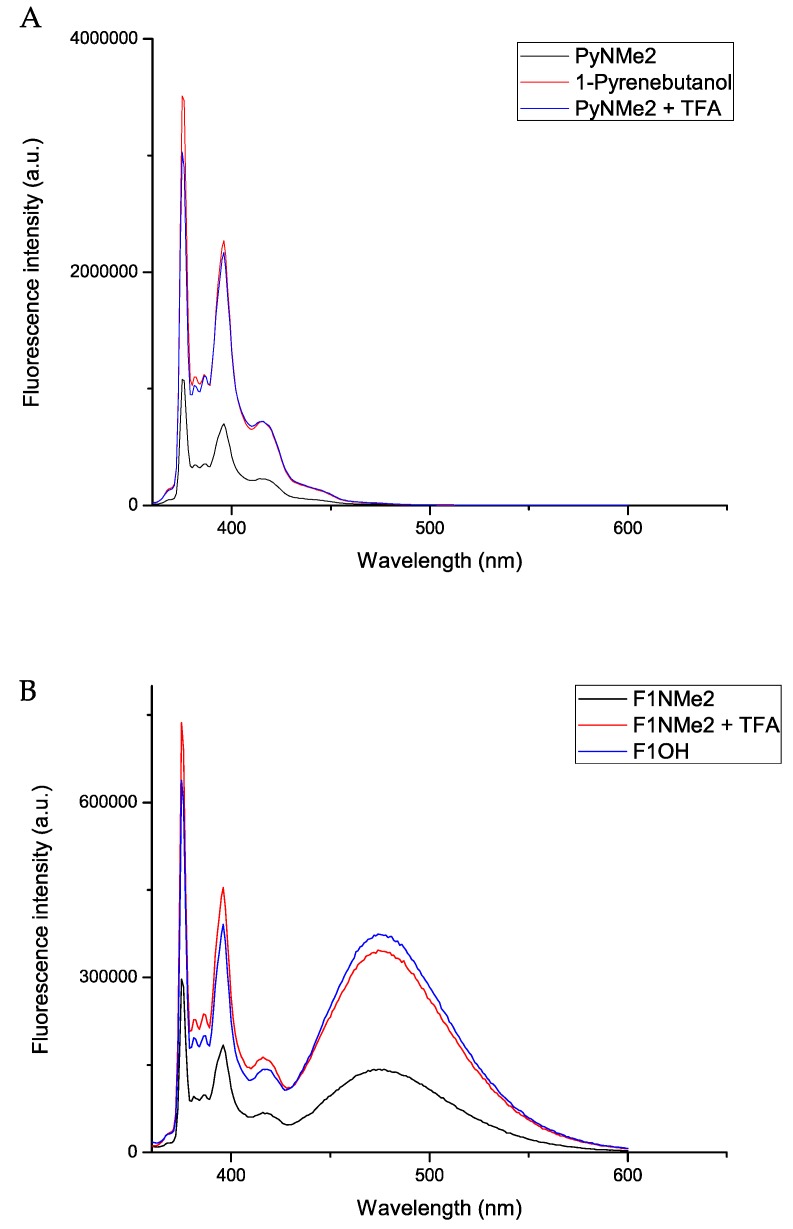
SSF analysis of (**A**) **PyNMe2** comparison with 1-pyrenebutanol and (**B**) **F1NMe2** derivative comparison with first generation alcohol derivative F1OH.

Initially, the amines **PyNMe2** and **F1NMe2** were studied by SSF and SPC. The first compound shows a lower emission in comparison with the respective alcohol 1-pyrenbutanol (Figure 2). The fluorescence of **PyNMe2** decreases to 28% in comparison with the alcohol derivative. The reduction of pyrene emission in SSF analysis is due to the quenching of its fluorescence by the amine pendant; this phenomenon is very well-known and occurs via a PET mechanism [35].

**Figure 3:**
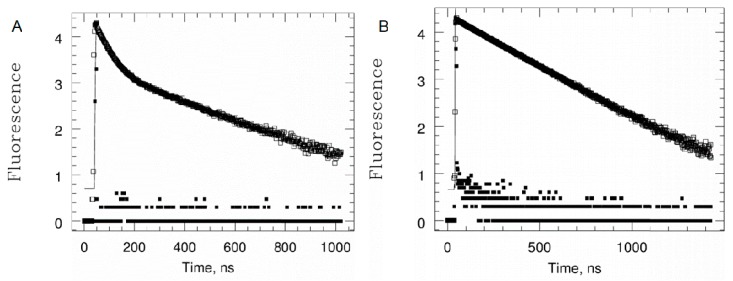
SPC analysis of **PyNMe2** recorded at 344 nm before (**A**) and after (**B**) TFA addition.

Similarly, the time-resolved analysis on PyNMe2 shows two different decays: a longer lifetime, correspondingly 200 ns for the alcohol, due to traces of pyrenbuthanol, and a second decay relative to the amine, equal to 36 ns (Figure 3). The absence of a shorter lifetime for the amine suggests that this phenomenon is a static quenching, with the presence of a ground-zero complex between pyrene and tertiary amine. Then, by adding one equivalent of trifluoroacetic acid to the amine solution, the emission of **PyNMe2** rises and the lifetime of 36 ns prolongs to 200 ns, which is typical for a pyrenebutane unquenched system. The increase of fluorescence and lifetime is related to the protonation of the nitrogen atom. Here, the quenching process involves the free electron pair of the nitrogen, which becomes unavailable in the presence of acid medium, due to its basic behavior. The acidification of the solution does not allow the tertiary amine to quench the pyrene fluorescence.

A similar study has been performed on F1 derivates. In Figure 2B, the SSF analysis of the Fréchet-type dendrons are shown. It can be easily seen that the **F1NMe2** derivative is characterized by a lower emission, in comparison with the analogue alcohol **F1OH**. All the decays have been recorded at a wavelength of 344 nm, which correspond to the S_0_–S_1_. The SPC analysis presented in this work has been done using the Model Free Analysis. [16,22]. It was observed (Table 1) that both dendrons display a similar <*k*>, having a relevant difference only in the T_E0_ value, which represents the lifetime of the excimer.

By adding one equivalent of TFA, the emission intensity increases (Figure 2). The addition of TFA to the solution containing the dendritic compound is expected to result in the protonation of the tertiary amine, yielding the adduct F1NMe2H^+^TFA. Surprisingly, the emission of the protonated **F1NMe2** is higher than the fluorescence of the Fréchet-type dendron bearing an alcohol group (**F1OH**). The higher photophysical performance of the protonated F1NMe2H^+^TFA^−^ is confirmed by the extended excimer lifetime, always in comparison with F1OH. An interesting result is that the <*k*> value remains almost constant for all the F1 systems; this fact means that the presence of amine or hydroxyl groups have no relevant influence on the excimer formation for this kind of system.

Thanks to these studies on the model compounds, we could expect an influence of the nitrogen atom on the photophysical properties of the cyclen–pyrene dendrimers, based on their quenching abilities shown in these preliminary analyses.

#### 2.1.2. Fluorescence of the Pyrene Dendrimers

Our work is focused on the study of the photophysical properties of three different dendrimers, labeled with four (**CyPy4**), eight (**CyPy8**) and sixteen (**CyPy16**) pyrene units, respectively, in order to investigate the influence of the potential quencher abilities of the nitrogen atoms present in the cyclen core. The synthesis of these dendrimers has been described in a previous publication [36]. The dendrimers were studied in different solvents (THF, DMF and DMSO), having different polarity and viscosity, since these parameters are able to influence the fluorescence properties of pyrene. As we did for the model compounds, SSF and SCP analysis were performed with these constructs. 

**Figure 4:**
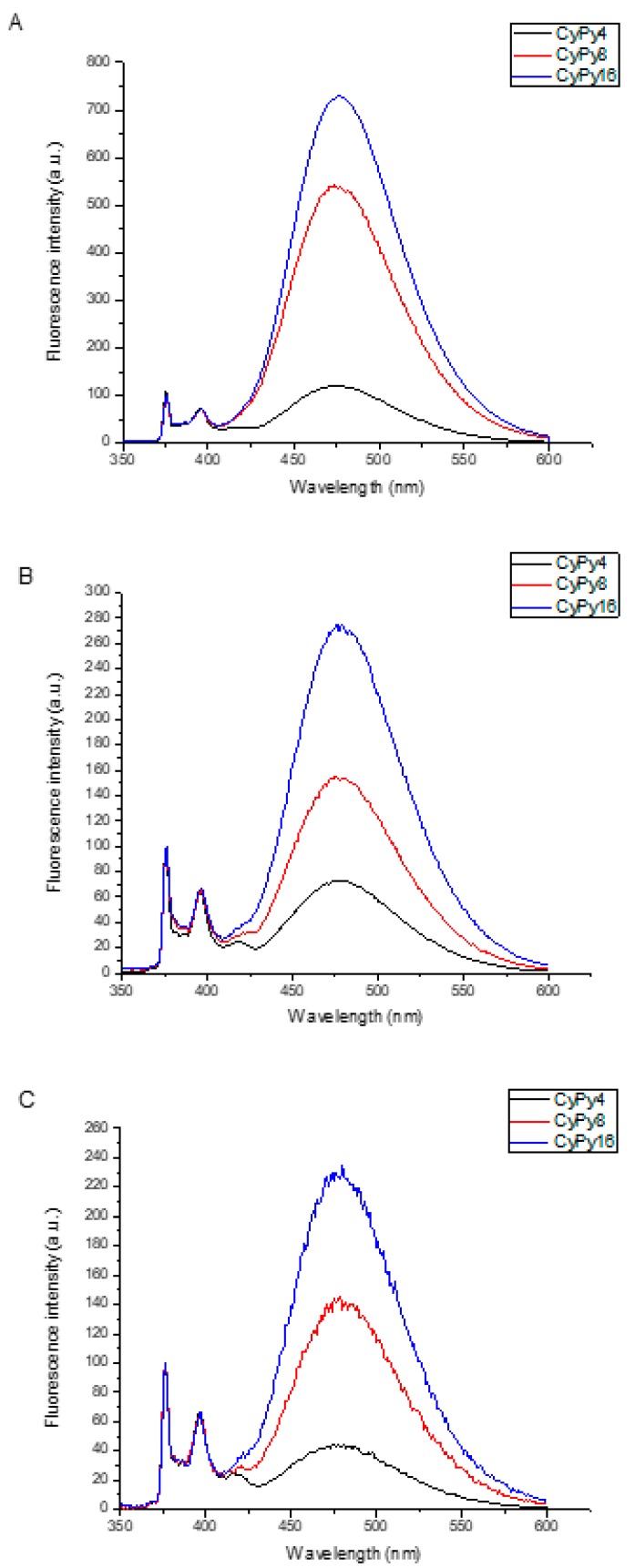
Steady-state fluorescence spectra of dendrimers **CyPy4**, **CyPy8**, **CyPy16** in (**A**) THF, (**B**) DMF, (**C**) DMSO.

The SSF analysis of the dendrimers **CyPy4**, **CyPy8** and **CyPy16** are shown in Figure 4. In the plots, independently of the kind of solvent, an increase of excimer formation passing from **CyPy4** to **CyPy16** can be clearly detected, which is due to the increase in the local content of pyrene in the dendrimers. In other words, the augment of pyrene’s moieties in these molecules results in a higher probability that each pyrene interacts with another pyrene molecule to form an excimer.

By changing the solvent, it is possible to detect several differences between the dendrimers. The first investigated solvent was THF. In this case, a huge excimer formation is observed for **CyPy16** dendrimer: this affirmation is proved by the high I_E_/I_M_ ratio equal to 8.69. This ratio compares the intensity of the monomer emission (area between 373 and 379 nm) to that of excimer emission (area between 500 and 530 nm), giving information about how much excimer is formed. By decreasing the amount of pyrene to 8 (**CyPy8**) and 4 (**CyPy4**), this ratio decreases to 6.32 and to 1.37, respectively. This reduction is not directly proportional to the number of pyrene units in the dendritic constructs, probably due to steric hindrance, which alters the diffusion ability of the dendrimer branches. In the same solvent, SPC analysis confirms the obtained data in SSF. **CyPy16** is the dendrimer with higher <*k*>, equal to 0.271, in comparison with the 8-pyrene and the 4-pyrene systems, whose <*k*> is 0.226 and 0.047, respectively. The higher value represents the improved capacity to form excimer. Similarly, the values of <**τ**> decrease with the increasing number of pyrenes in the dyads. In fact, the average monomer lifetime decrease from 19.3 ns for **CyPy4** to 3.6 ns for **CyPy16**, passing through 4.3 ns for **CyPy8**. These data mean that the pyrene moieties remain longer in the excited state for CyPy4 than in the other dendrimers. These data can be explained by the lower local concentration of fluorescent probe, thereby decreasing the probability to interact with others pyrene units.

Afterwards, we decided to verify how polarity would affect the photophysical properties of the dendrimers. For that, we chose DMF as solvent, which has a higher polarity than THF (relative polarity for THF is 0.207 while for DMF is 0.386) [37]. As in the previous solvent, in this case, the SSF analysis shows the peaks relative to the monomer and excimer emission for all the derivatives. Similarly to the results obtained in THF, the excimer emission intensities are directly related to the number of pyrene units present in the molecules. **CyPy16** has the higher excimer emission, followed by **CyPy8** and finally, by **CyPy4**. The parameter I_E_/I_M_ confirms this affirmation: the **CyPy4** ratio is equal to 0.77, and **CyPy8** and **CyPy16** ratios to 1.63 and 3.33, respectively. On the other hand, it is interesting to make a comparison with the data obtained in the less polar and less viscous solvent [38]. All the ratios are lower in DMF, compared to those observed in THF: for **CyPy4** 1.37 vs 0.77, for **CyPy8** 6.32 vs 1.63 and for **CyPy16** 8.69 vs 3.33. The excimer emission reduction is attributed to the higher viscosity of DMF, which makes the approach of pyrene units in the dendrimer more difficult. SPC analyses were performed in DMF and the results show a dualistic effect for the lifetime of the photoactive dyes. At first, the higher viscosity of the solvent should increase the lifetime of the pyrene units in the excited state in comparison with the ones obtained in THF. This affirmation finds confirmation in the case of the bulky **CyPy8** and **CyPy16**, for both systems <**τ**> is higher in DMF, as shown in Table 2B. Usually, for both systems, the lifetime is 1 ns larger in DMF than in THF. For the dendrimer **CyPy4**, this trend is not found, due to the higher mobility of the periphery. In fact, DMF is a high-polarity solvent which has low compatibility with non-polar pyrenes. In order to reduce the interaction with DMF, the pyrene-containing branches coil towards the dendrimer core, allowing pyrenes to be closer to each other. This compact configuration involves a higher possibility to have pyrene–pyrene intramolecular interactions and explains why the lifetime for **CyPy4** is lower. It is important to consider the <k> values for these dendrimers in DMF: **CyPy4** shows the lower value, equal to 0.099, instead **CyPy8** and **CyPy16** have <k> values of 0.125 and 0.206, respectively. These values usually change with the increase of the number of pyrenes in the compound and decrease of the viscosity of the solvent.

Finally, we used DMSO as a solvent, which shows a higher polarity and viscosity if compared with THF and DMF. For these analyses, steady-state and single-photon counting studies do not show relevant differences with respect to the results discussed before in DMF. In fact, the ratio I_E_/I_M_ keeps decreasing, due to the higher viscosity that makes it more difficult for the pyrene moieties to come into contact in the excited state to form an excimer. For instance, the ratio is equal to 0.88 for **CyPy4** in DMF and decreases to 0.55 in the case of DMSO. Instead, SSC analyses display <**τ**> values almost similar to those in DMF and DMSO, due to the dual effect of the high viscosity (which decreases the mobility of the pyrene moieties, increasing the lifetime) and high polarity (**CyPy4** labelling results closer than in THF, decreasing the lifetime). Even in this solvent, it is worth mentioning that the registered <k> values, which are always similar for the two polar solvents: for **CyPy4** this value is equal to 0.078, for **CyPy8** is 0.123 and for **CyPy16** is 0.186. As mentioned before, these values are proportional to the number of pyrene units present in the molecule. Therefore, in CyPy4, the pyrene units are closer to each other since it is a small dendrimer so that the encounter of pyrenes with each other is less affected by the polarity and viscosity of the solvent. However, in CyPy8, the pyrenes are further away from each other and it is very difficult for them to encounter on another by diffusion in a more viscous solvent. This tendency can be easily observed in high-generation dendrimers but not in CyPy4.

Finally, an interesting observation was made by considering the *f* values relative to the excimer formation. Almost independently from the dendrimer generation or the solvent, most of the excimer emission is due to diffusive processes. The main difference is found in the type of excimer formed; for the higher generations **CyPy8** and **CyPy16**, the most abundant excimer showed a longer lifetime (**τ** D), meanwhile, for **CyPy4**, the obtained excimer has a shorter lifetime (**τ**_EO_).

#### 2.1.3. Shorter Lifetime for CyPy4

The SPC analysis of dendrimer **CyPy4** shows interesting data (Table 3): in all the investigated solvents, shorter lifetimes are found. The lifetime is slightly longer in the case of THF (2.44 ns) than for DMF (0.51 ns) and DMSO (0.57 ns). Furthermore, by checking the data, it is clear that the phenomenon, relative to this short lifetime, is the dominant one related to the photophysical behavior of this dendrimer, which is very sensitive to the polarity of the solvent showing a charge transfer character. In all the employed solvents, almost 50% of the pyrene undergoes to this process.

Additional investigation is of merit to gain a deeper insight in this phenomenon, which is relatively difficult due to the short time windows—equal to 2 ns or lower. According to the data collected so far, we can affirm that this process is strongly influenced by the polarity of the solvent. In fact, the analysis shows the longest lifetime is observed in the less polar solvent (THF) and the shortest one (≈0.5 ns) in the more polar solvents. In DMF and DMSO, the data are similar due to the same reason that was explained in the previous paragraph.

### 2.2. Quenching Studies

The main objective of this work is to analyze the quenching phenomenon due to the nitrogen atoms present in the dendrimer core. The cyclen core is constituted by tertiary amines, which are well known quenchers of pyrene fluorescence. On the other hand, ammonium salts are not able to interact with the luminescent probe, because the nitrogen lone pair is not available. It means that amine quenching of pyrene is reversible by adding acid into the solution containing the dye and the tertiary amine.

The dendrimers studied in this work are analogues of the naphthalene ones previously studied, which have a cyclam unit (1,4,8,11-tetraazacyclotetradecan) as their core [39]. In that report, it is clarified that the core, which contains nitrogen atoms, is able to quench the naphthalene fluorescence and the reversibility of this phenomenon was shown by making the nitrogen lone pair unavailable by complexation with bivalent metal ions.

For our work, we decided to prepare different solutions containing 2.5 × 10^−6^ M of dendrimer and different equivalents of trifluoroacetic acid—up to 3 equivalents in relation to the dendrimer. We used a strong acid in order to ensure the quantitative dissociation and, consequently, obtain the equivalent amount of H^+^ ions in the solution. The acidification of the solution containing the dendrimers, should result in the protonation of the tertiary amines present in the cyclen core: the tertiary amines have shown quenching properties in the aforementioned study with the model compounds PyNMe2 and F1NMe2. The addition of TFA to the dendrimer solution is likely to cause the inhibition of the intramolecular quenching properties of the amine due to the protonation of the lone pair of the nitrogen atom, as a consequence of the PET process and following the decrease of the pyrene fluorescence. The expected result is an increase of the pyrene emission due to reversed amine quenching by the solution acidification.

**Figure 5:**
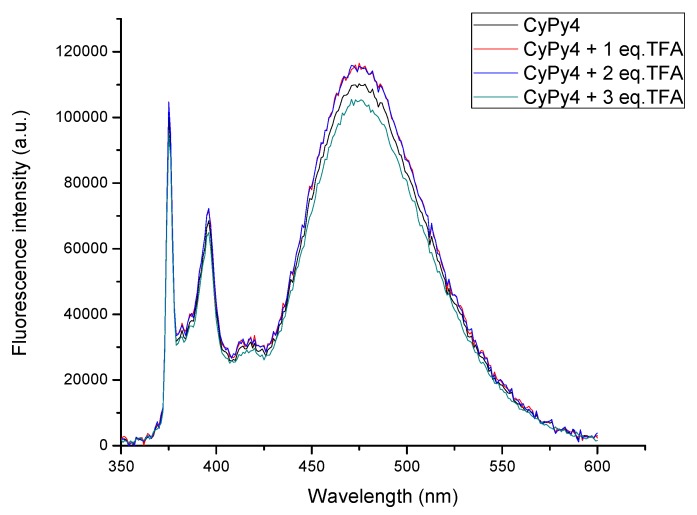
Steady-state **CyPy4** before and after the addition of 1 to 3 equivalents of TFA.

To study the influence of the acid on the fluorescence of the dendrimers, we focused on the I_E_/I_M_ ratio, obtained by SSF analysis, and on the <*k*> obtained by SPC analysis. To exemplify the result obtained by SSF, the study of **CyPy4** is presented in Figure 5. Unpredictably, there are no relevant variations in the emission spectrum of the dendrimer, even by addition of TFA equivalents. To have objective data to compare, the I_E_/I_M_ ratio is taken into account (Table 4). From the displayed data, all the dendrimers analyzed in THF have a similar emission independently of the acid equivalent present in the solution.

In THF, the ratios for **CyPy4** are close to 1.37, with differences attributable to the sample preparation instead of an actual effect of TFA. Similarly, the **CyPy8** ratio is constant and equal to 6.32, even considering only the dendrimer or the dendrimer in the presence of 3 equivalents of TFA. The same consideration can be applied to the **CyPy16** compound, which has a ratio close to 8.69. Comparing the SFC analysis without acid, THF results were relevantly different from those recorded in DMF and DMSO. Such differences were attributed to polarity and viscosity of the solvent. Surprisingly, similar results are obtained even for these solvents.

Taking into account the analysis in DMF, for **CyPy4**, the ratio is lower than in THF and it is equal to 0.88. This value does not change by adding TFA, thereby showing an ineffective outcome of the pyrene-labeled compound. Similarly, in these data, **CyPy8** does not seem to be affected by the addition of acid, keeping an I_E_/I_M_ ratio of 1.63. Finally, the analyses on **CyPy16** in the presence of TFA does not show any relevant change in the emission. In the previous dendrimers, this ratio remains constant and equal to 3.33.

Finally, we repeated the analysis in DMSO. As in the data recorded in THF and DMF, it is not possible to observe any relevant difference, even for this final solvent. In fact, the addition of TFA does not influence the dendrimers’ fluorescence for any of them. For **CyPy4**, the I_E_/I_M_ ratio remains constant for all the experiments, showing a value close to 0.55. In the case of **CyPy8**, the addition of acid is ineffective and the ratio is equal to 1.85. Finally, **CyPy16** exhibits a similar behavior and its ratio is registered as being consistently close to 3.02.

The quenching of pyrene fluorescence affects both the quantity of emitted light and the timing of the photophysical process. In the case of timing, quenching is a fast phenomenon, which increases the value of <*k*> by reversing the quenching, we should assist to a decrease of the rate constant for the photophysical processes. The values for these systems were calculated using the Model free analysis method.

**Figure 6:**
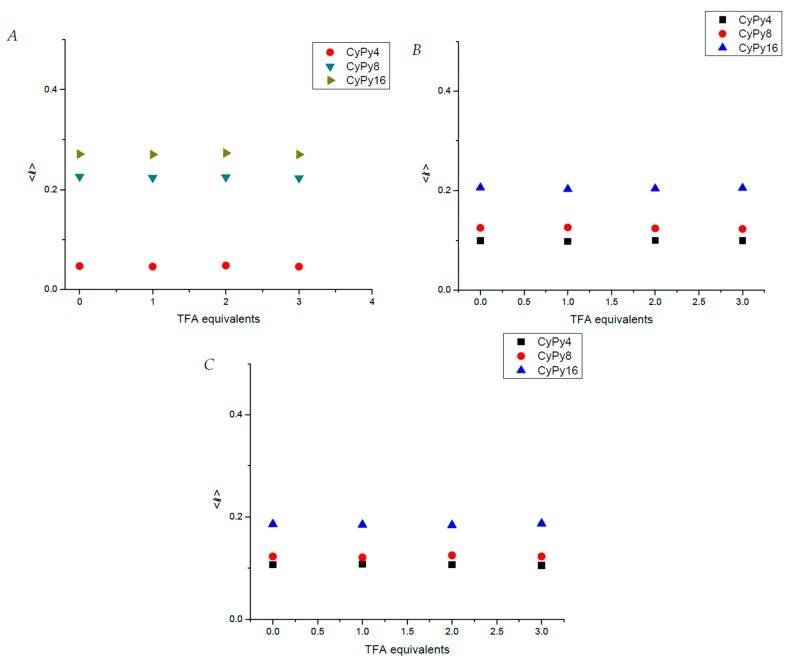
<k> values of pyrene-labeled dendrimers in (**A**) THF, (**B**) DMF, (**C**) DMSO, with different amounts of TFA.

The first study was performed in THF. In Figure 6, it is possible to observe that **CyPy4** has a lower <*k*> in comparison with the other dendrimers, due to the lower content of pyrene in the structure; for this compound <*k*> is equal to 0.047. By adding 1 equivalent of TFA, the expected result should be an increase in the rate constant, surprisingly, it does not change after the addition. Further addition of acid does not affect this value. For **CyPy8**, we can observe a higher <*k*> value, equal to 0.226. As seen before, adding TFA to the pyrene-labeled compound does not modify the <*k*> constant. Also, the same observation is made for **CyPy16**, whose average rate constant is equal to 0.247, independently from the presence or absence of acid.

Afterwards, the SPC analysis of the dendrimer was performed in DMF. The <*k*> of **CyPy8** and **CyPy16** decrease due to the higher viscosity of this solvent, which reduces the mobility of the branches. On the other hand, **CyPy4** exhibits an increase of the average rate constant due to the polarity, which forces pyrenes to get closer to the nearest nonpolar scaffold constituted by the core. For this compound, the <*k*> in DMF is equal to 0.099; after the addition of TFA equivalents, this value does not change. Concerning **CyPy8**, the measured <*k*> is equal to 0.125, which is lower than that observed in THF. The addition of acid is ineffective even for this compound, since the average rate constant does not change significantly. Next, the SPC studies on **CyPy16** show an analogue behavior with respect to **CyPy8**; the <*k*> decreases in comparison to that measured in THF (0.206) but it is not modified by the presence of various equivalents of TFA.

Finally, our analysis was achieved using DMSO as a solvent. The data obtained in this solvent confirm the infectiveness of the acid addition to the solution. In fact, for all the analyzed compounds, the <*k*> value does not change notably. Considering **CyPy4**, the average rate constant measured is equal to 0.107, independently from the presence of TFA. For **CyPy8**, the rate constant is equal to 0.123, while for **CyPy16**, it is 0.186.

According to the observation discussed in this paragraph, there is no clear evidence of pyrene fluorescence quenching due to the presence of tertiary amine in the core of the dendrimer constituted by a cyclen unit. These results are unexpected, since there are no reports about this unusual behavior of the nitrogen–pyrene pair.

## 3. Materials and Methods

*Chemicals.* All the reagents involved in the synthesis were purchased from Aldrich (Toluca, Estado de México) and used as received. The solvents used in the reactions were purified by simple distillation. The synthetic scheme for the model compounds, ^1^H and ^13^C NMR spectra of compounds are in the Supplementary Materials.

*Steady-State Fluorescence.* PTI spectrofluorometer equipped with a xenon arc lamp was used to acquire the steady-state fluorescence (SSF) spectra of the dendritic and model compounds. These spectra were recorded with excitation at 344 nm and scanning the emission from 350 to 600 nm with excitation and emission slit widths of 2 and 1 nm, respectively. After the preparation of solutions containing 2.5 × 10^−6^ M of pyrene-labeled compound, such solutions were outgassed with a gentle flow of N_2_ gas for 25 min before acquiring the SSF spectrum, in order to avoid the presence of oxygen. A similar pre-treatment was done for the solution containing TFA and the pyrene-labeled compounds. 

*Time-Resolved Fluorescence.* The lifetime fluorescence decays of pyrene-labeled dendrimers and model compounds were acquired using an IBH TCSPC spectrofluorometer equipped with a NanoLED-340 laser. All decays were collected with an excitation wavelength of 344 nm over 1024 channels. Using a time per channel of 2.04 ns channel^−1^, the monomer fluorescence decay was collected at an emission wavelength of 375 nm, using a 370 nm cutoff filter. The excimer fluorescence decays were acquired at 510 nm with a 495 nm cutoff filter, using a time per channel of 1.02 ns channel^−1^. The instrument response function was collected using a LUDOX dispersion in water with a maximum peak at 344 nm. The reported decays are presented as Log of Counts on y axis and Time in x axis. The analysis of the decays has been carried out following the equation presented in previous work [16,22].

## 4. Conclusions

In this work, we investigated the photophysical behavior of three Fréchet-type dendrimers, having cyclen as the core, labeled with different numbers of pyrene units in the periphery. Due to the presence of tertiary amine in the core, we studied the possibility of a quenching process of pyrene fluorescence, using SSF and SPC analysis in different solvents. The investigated solvents were THF, DMF and DMSO, which have different viscosities and polarities. To verify the effective interaction between pyrene and the amine, two model compounds were synthesized, **PyNMe2** and **F1NMe2**. These compounds were analyzed in THF, showing the typical quenching behavior of a tertiary amine on pyrene, and the possibility to revert this process by the addition of TFA. In this way, ammonium salts are formed, showing behaviors similar to the respective alcohol derivates (PyOH and F1OH). Dendrimers **CyPy4**, **CyPy8** and **CyPy16** were analyzed by SSF, obtaining the I_E_/I_M_ ratios, and SPC, focusing on <**τ**> and <*k*>. I_E_/I_M_ ratios increase depending on the number of pyrenes present in the dendritic molecules and decrease depending on the viscosity of the solvent. The <*k*> for the compounds decrease for **CyPy8** and **CyPy16** with the increase of the viscosity of the solvents; however, it increases for **CyPy4** due to the higher polarity, which causes intramolecular aggregation.

It was observed that the addition of TFA to the dendrimer solution has no effect on SSF and SPC analysis. In fact, the I_E_/I_M_ ratio and the <*k*> value remain constant for all the compounds independently from the presence or absence of trifluoroacetic acid. This result was obtained in all the studied solvents. These behaviors show the absence of quenching by tertiary amine on pyrene fluorescence, resulting in an unusual and interesting phenomenon, which deserves further investigation.

## Figures and Tables

**Table:** 

	a_1_	τ_1_	a_2_	τ_2_	a_3_	τ_3_	a_M_	τ_M_	<τ>	<k>
**F1OH**	0.553	16.39	0.280	32.17	0.098	73.54	0.068	200	27.2	0.031
**F1NMe2**	0.546	15.70	0.322	30.69			0.130	56	21.3	0.029
**F1NMe2H+TFA^−^**	0.459	18.71	0.312	38.32	0.148	72.53	0.080	200	34.0	0.024

**Table:** 

	$f_{Ediff}^{E0}$	$f_{E}^{E0}$	τ_EO_
**F1OH**	0.992	0.007	45.78
**F1NMe2**	0.976	0.236	35.12
**F1NMe2H+TFA^−^**	0.992	0.007	50.31

τ**_x_** = decay time; **a_x_** = pre-exponential related to τ_x_; **a_M_** and τ**_M_** are related to the monomer (residual pyrenebuthanol); <τ> = number-average lifetime; <k> = excimer rate formation; τ**_EO_** = decay time of excimer formed by properly stacked pyrene dimers; $f_{Ediff}^{E0}$ = molar fraction of excimer formed by properly stacked pyrene dimers formed by diffusion with decay time equal to τ**_EO_**; $f_{E}^{E0}$ = molar fraction of excimer formed by properly stacked pyrene dimers formed in basal state with decay time equal to τ**_EO_**.

**Table:** 

A) THF	a_1_	τ _1_	a_2_	τ_2_	a_3_	τ_3_	a_M_	τ_M_	a_MS_	τ_S_	<τ >	<k>
**CyPy4**	0.138	6.52	0.197	15.66	0.052	66.59	0.030	200	0.581	2.44	19.3	0.047
**CyPy8**	0.768	2.11	0.175	6.22	0.046	33.92	0.009	200			4.3	0.226
**CyPy16**	0.724	1.62	0.225	5.45	0.038	30.16	0.011	200			3.6	0.271

**Table:** 

	$f_{Ediff}^{E0}$	$f_{E}^{E0}$	τ_EO_	$f_{Ediff}^{D}$	$f_{E}^{D}$	τD	$a_{E}^{s}$	τs
**CyPy4**	0.653	0.345	46.12	0	0	35	0.0052	0.51
**CyPy8**	0.149	0	38.25	0.797	0.053	50.73		
**CyPy16**	0.349	0.005	37.86	0.605	0.040	54.58		

**Table:** 

B) DMF	a_1_	τ _1_	a_2_	τ _2_	a_3_	τ _3_	a_M_	τ _M_	a_MS_	τ _S_	<τ>	<k>
**CyPy4**	0.200	2.41	0.118	12.57	0.045	32.74	0.029	163	0.605	0.51	9.5	0.099
**CyPy8**	0.740	3.11	0.162	10.99	0.074	44.80	0.022	163			7.6	0.125
**CyPy16**	0.627	2.10	0.331	6.92	0.033	31.78	0.008	163			4.7	0.206

**Table:** 

	$f_{Ediff}^{E0}$	$f_{E}^{E0}$	τ_EO_	$f_{Ediff}^{D}$	$f_{E}^{D}$	τD	$a_{E}^{s}$	τs
**CyPy4**	0.653	0.345	46.12	0	0	35	0.0052	0.51
**CyPy8**	0.149	0	38.25	0.797	0.053	50.73		
**CyPy16**	0.349	0.005	37.86	0.605	0.040	54.58		

**Table:** 

C) DMSO	a_1_	τ_1_	a_2_	τ_2_	a_3_	τ_3_	a_M_	τ_M_	a_MS_	τ_S_	<τ>	<k>
**CyPy4**	0.523	1.48	0.325	6.97	0.123	44.04	0.027	130	0.298	0.86	8.74	0.106
**CyPy8**	0.594	3.24	0.300	9.43	0.090	30.38	0.014	130			7.62	0.123
**CyPy16**	0.595	3.16	0.363	6.44	0.036	24.89	0.003	130			5.15	0.186

**Table:** 

	$f_{Ediff}^{E0}$	$f_{E}^{E0}$	τ _EO_	$f_{Ediff}^{D}$	$f_{E}^{D}$	τ_D_	$a_{E}^{s}$	τs
**CyPy4**	0.706	0.293	33.81	0	0	35	0.0	0.86
**CyPy8**	0.393	0.022	33.58	0.514	0.068	43.14		
**CyPy16**	0.525	0.005	33.55	0.404	0.064	44.21		

**τ_x_** = decay time; **a_x_** = pre-exponential related to **τ**_x_; **a_M_** and **τ _M_** are related to the monomer (residual pyrenebuthanol); **a_MS_** and **τ _MS_** are related short time decay; <**τ**> = number-average lifetime; <k> = excimer rate formation; **τ_EO_** = decay time of excimer formed by properly stacked pyrene dimers; $f_{Ediff}^{E0}$ = molar fraction of excimer formed by properly stacked pyrene dimers formed by diffusion with decay time equal to **τ _EO_**; $f_{E}^{E0}$ = molar fraction of excimer formed by properly stacked pyrene dimers formed in basal state with decay time equal to **τ _EO_**; **τ_D_** = decay time of the excimer formed by improperly stacked pyrene dimers; $f_{Ediff}^{D}$ = molar fraction of excimer formed by improperly stacked pyrene dimers formed by diffusion with decay time equal to **τ _D_**; $f_{E}^{D}$ = molar fraction of excimer formed by improperly stacked pyrene dimers formed in basal state with decay time equal to **τ_D_**; $a_{E}^{s}$ = molar fraction of excimer formed by pyrene showed short decay time; **τ**s = short decay time.

**Table:** 

	a_1_	τ_1_	a_2_	τ_2_	a_3_	τ _3_	a_M_	τ_M_	a_MS_	τ_S_	<τ>	<k>
THF	0.138	6.52	0.197	15.66	0.052	66.59	0.030	200	0.581	2.44	19.3	0.047
DMF	0.200	2.41	0.118	12.57	0.045	32.74	0.029	163	0.605	0.51	9.5	0.099
DMSO	0.523	1.48	0.325	6.97	0.123	44.04	0.027	130	0.298	0.86	8.74	0.106

**Table:** 

	$f_{diff}^{}$	$f_{free}^{}$	$f_{agg}^{D}$	$f_{s}^{}$
THF	0.367	0.028	0.055	0.550
DMF	0.305	0.025	0.162	0.508
DMSO	0.387	0.014	0.235	0.364

**τ_x_** = decay time; **a_x_** = pre-exponential related to **τ**_x_; **a_M_** and **τ _M_** are related to the monomer (residual pyrenebuthanol); **a_MS_** and **τ _MS_** are related short time decay; **<τ>** = number-average lifetime; **<k>** = excimer rate formation; $f_{diff}^{}$ = molar fraction of excimer formed by diffusion ; $f_{free}^{}$ = molar fraction of the monomer; $f_{agg}^{D}$ = molar fraction of excimer formed by improperly stacked pyrene dimers formed in basal state; ***f*_s_** = molar fraction of pyrene with decay time equal to **τ_s_**; **τ**_s_ = short decay time.

**Table:** 

A) THF	0 eq	1 eq	2 eq	3 eq
**CyPy4**	1.379	1.385	1.383	1.370
**CyPy8**	6.323	6.312	6.340	6.345
**CyPy16**	8.693	8.689	8.694	8.691

**Table:** 

B) DMF	0 eq	1 eq	2 eq	3 eq
**CyPy4**	0.883	0.882	0.883	0.882
**CyPy8**	1.630	1.629	1.631	1.627
**CyPy16**	3.330	3.323	3.318	3.314

**Table:** 

C) DMSO	0 eq	1 eq	2 eq	3 eq
**CyPy4**	0.555	0.554	0.555	0.554
**CyPy8**	1.857	1.873	1.872	1.873
**CyPy16**	3.024	3.038	3.033	3.037

## Supplementary Materials

The following are available online at https://www.mdpi.com/1420-3049/24/22/4083/s1. Scheme S1: Synthetic scheme for the model compounds PyNMe2 and F1NMe2; Figures S1–S4: 1H-NMR and 13C-NMR and spectra of compounds; Figures S5–S17: SPC of compounds; Figures S18–S26: SSF of compounds.
