# Supplementary material for: Unusual Fluorescence Behavior of Pyrene-Amine Containing Dendrimers
Source: Molecules. 2019 Nov 12;24(22):4083. doi: 10.3390/molecules24224083 (PMC6891302; doi:10.3390/molecules24224083)
Supplement: Supplementary file 1 [file molecules-24-04083-s001.pdf]

Supporting information

“Unusual fluorescence behavior of pyrene-amine containing dendrimers”

Andrea Ruiu *, Mireille Vonlanthen, Sandra M. Rojas-Montoya, Israel González-Méndez,

and Ernesto Rivera *

Instituto de Investigaciones en Materiales, Universidad Nacional Autónoma de México, Circuito Exterior Ciudad Universitaria, C.P. 04510, Ciudad de México, México.

INDEX

1. Synthetic Procedures	S3-S6
2. **Figure S1**. ^{1}H-NMR spectra of **PyNMe2**	S7
3. **Figure S2**. 13C-NMR spectra of **PyNMe2**	S7
4. **Figure S3**. ^{1}H-NMR spectra of **F1NMe2**	S8
5. **Figure S4**. ^{13}C-NMR spectra of **F1NMe2**	S8
6. **Figure S5-S8**. SPC of control compounds	S9-S12
7. **Figure S9-S11**. SPC of cyclen dendrimers in THF	S13-S15
8. **Figure S12-S14**. SPC of cyclen dendrimers in DMF	S16-S18
9. **Figure S15-S17**. SPC of cyclen dendrimers in DMSO	S19-S21
10. **Figure S18-S20**. SSF of cyclen dendrimers in THF: Quenching studies	S22-S24
11. **Figure S21-S23**. SSF of cyclen dendrimers in DMF: Quenching studies	S25-S27
12. **Figure S24-S26**. SSF of cyclen dendrimers in DMSO: Quenching studies	S28-S30
13. References	S31

Synthetic Procedures

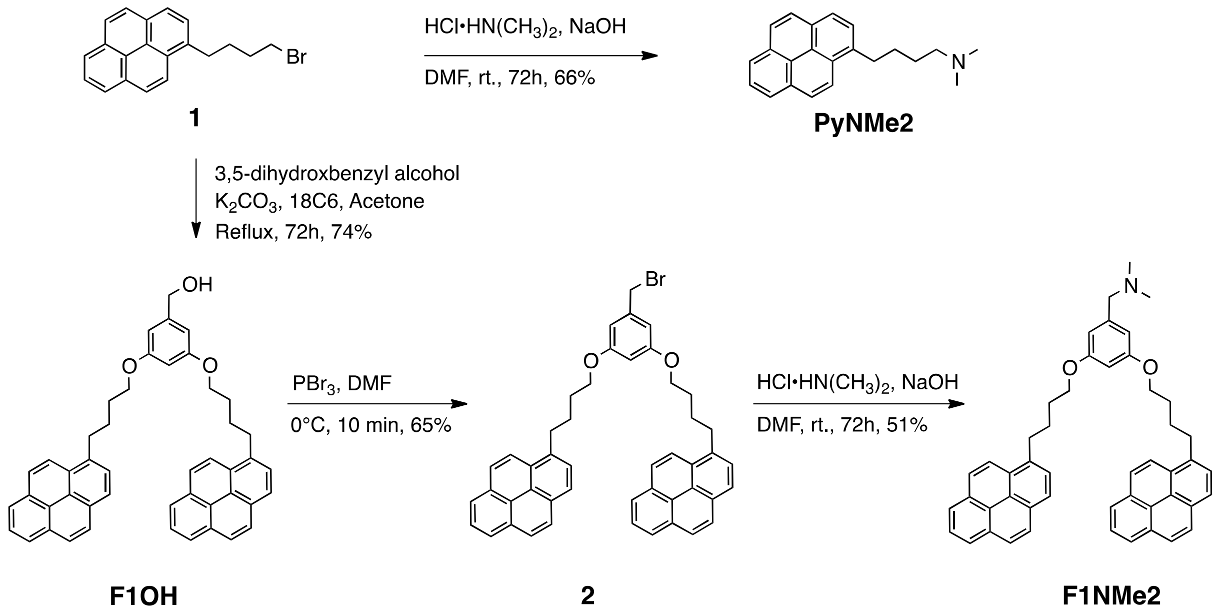

Scheme S1: Synthetic scheme for the model compounds **PyNMe2** and **F1NMe2**.

1-(4-Bromobutyl)pyrene (**1**)

Compound **1** was synthesized as previously reported[1]. The product was obtained as a white solid (2.190 g, 90%). ^{1}H NMR (δ ppm, 300 MHz, $CDCl_3$): 8.27-7.86 (m, 9H, CH Ar_{py}), 3.44 (t, 2H, ^3J = 6.3, CH_2), 3.38 (t, 2H, ^3J = 7.2, CH_2), 2.13-1.94 (m, 4H, CH_2).

N,N-dimethyl 4(pyren-1-yl)butan-1-amine (**PyNMe2**)

A solution of 1-(4-Bromobutyl)pyrene **1** (84 mg, 0.25 mmol) in DMF (3 ml) was prepared. Dimethylamine hydrochlorid (204 mg, 2.5 mmol) and sodium hydroxyde (100 mg, 2.5 mmol) were added to the reaction mixture and it was stirred at room temperature for 48h. Another portion of dimethylamine hydrochloride (102 mg, 1.25 mmol) was added and the reaction was reacted further for 24h at room temperature. The reaction mixture was evaporated and the crude

product was taken in dichloromethane and washed with water. The organic phase was dried over $MgSO_4$ and evaporated. The product was purified by column chromatography (dichloromethane:hexane 1:1 ; dichloromethane ; dichloromethane:methanol 97:3) to give the desired product **PyNMe2** as a light brown sticky solid (50 mg, 66 %).

^{1}H NMR (ppm, 400 MHz, $CDCl_3$): 8.26 (d, 1H, ^3J = 9.3, pyrene), 8.18-8.09 (m, 4H, pyrene), 8.02-7.97 (m, 3H, pyrene), 7.86 (d, 1H, ^3J = 7.8, pyrene), 3.38 (t, 2H, ^3J = 7.6, Py-CH_2), 3.42-3.38 (t, 2H, ^3J = 7.6, N-CH_2), 2.39 (s, 6H, N(CH_3)$_2$), 1.94-1.86 (m, 2H, CH_2-CH_2), 1.82-1.74 (m, 2H, CH_2-CH_2). ^{13}C NMR (ppm, 100 MHz, $CDCl_3$): 136.2 (C), 131.5 (C), 131.0 (C), 130.0 (C), 128.7 (C), 127.6 (CH), 127.44 (CH), 127.37 (CH), 126.8 (CH), 126.0 (CH), 125.2 (C), 125.1 (C), 125.0 (CH), 124.94 (CH), 124.87 (CH), 123.4 (CH), 59.0 (CH2), 44.4 (CH3), 33.3 (CH2), 29.3 (CH2), 26.4 (CH2). DART-MS: Calculated for $C_{22}H_{23}N$ 301.18, found 302.

First generation dendron (**F1OH**)

Compound **F1OH** was synthesized as previously reported [1]. The product was obtained as a white solid (0.980 g, 72%). ^{1}H NMR (δ ppm, 400 MHz, $CDCl_3$): 8.29-7.85 (m, 18H, CH Ar_{py}), 6.48 (d, 2H, ^4J = 2.1, ArH_o), 6.36 (t, 1H, ^4J = 2.1, ArH_p), 4.58 (d, 2H, ^3J = 3.8, CH_2 benzylic), 3.97 (t, 4H, ^3J = 5.8, O-CH_2), 3.39 (t, 4H, ^3J = 7.6, Py-CH_2), 2.10-1.88 (m, 8H, CH_2).

Brominated first generation dendron (**3**)

Compound **3** was synthesized according to the procedure previously reported by us [2]. The product was obtained as a white solid (144 mg). Yield: 65%. ^{1}H NMR (ppm, 400 MHz, $CDCl_3$): 8.28 (d, 2H, ^{3}J = 9.3, pyrene), 8.16-8.08 (m, 8H, pyrene), 8.02-7.95 (m, 6H, pyrene), 7.88 (d, 2H, ^{3}J = 7.8, pyrene), 6.50 (d, 2H, ^{4}J = 2.2, ArH_o), 6.36 (t, 1H, ^{4}J = 2.2, ArH_p), 4.36 (s, 2H, ArCH_2-Br), 3.98 (t, 4H, ^{3}J = 6.2, O-CH_2), 3.41 (t, 4H, ^{3}J = 7.6, Py-CH_2), 2.08-1.90 (m, 8H, CH_2-CH_2).

N,N-dimethyl first generation dendron (**F1NMe2**)

A solution of compound **3** (66 mg, 0.08 mmol) in DMF (3 ml) was prepared. Dimethylamine hydrochlorid (70 mg, 0.86 mmol) and sodium hydroxyde (34 mg, 0.86 mmol) were added to the reaction mixture and it was stirred at room temperature for 48h. Another portion of dimethylamine hydrochlorid (35 mg, 0.43 mmol) was added and the reaction was reacted further for 24h at room temperature. The reaction mixture was evaporated and the crude product was taken in dichloromethane and washed with water. The organic phase was dried over $MgSO_4$ and evaporated. The product was purified by column chromatagraphy (dichloromethane:hexane 1:1 ; dichloromethane ; dichloromethane:methanol 97:3) to give the desired product **F1NMe2** as a light brown sticky solid (30 mg, 51 %).

^{1}H NMR (ppm, 400 MHz, $CDCl_3$): 8.28 (d, 2H, ^{3}J = 9.3, pyrene), 8.16-8.07 (m, 8H, pyrene), 8.02-7.95 (m, 6H, pyrene), 7.87 (d, 2H, ^{3}J = 7.8, pyrene), 6.50 (d, 2H, ^{4}J = 1.9, ArH_o), 6.36 (t, 1H, ^{4}J = 1.9, ArH_p), 4.00 (t, 4H, ^{3}J = 6.2, O-CH_2), 3.42-3.38 (m, 6H, Py-CH_2, ArCH_2-N), 2.28 (s, 6H, N(CH_3)$_2$), 2.06-1.91 (m, 8H, CH_2-CH_2). ^{13}C NMR (ppm, 100 MHz, $CDCl_3$): 160.3 (C), 136.7 (C), 131.5 (C), 131.0 (C), 129.9 (C), 128.7 (C), 127.6 (CH), 127.4 (CH), 127.3 (CH),

126.7 (CH), 125.9 (CH), 125.2 (C), 125.1 (C), 125.0 (CH), 124.9 (CH), 124.8 (CH), 123.5 (CH), 107.7 (CH), 100.7 (CH), 67.9 (CH2), 64.3 (CH2), 45.1 (CH3), 33.3 (CH2), 29.4 (CH2), 28.4 (CH2).

Cyclen dendrimer of generation zero **(CyPy4)** first generation (**CyPy8**) and second generation (**CyPy16**) were synthesized as previously reported by us [3].

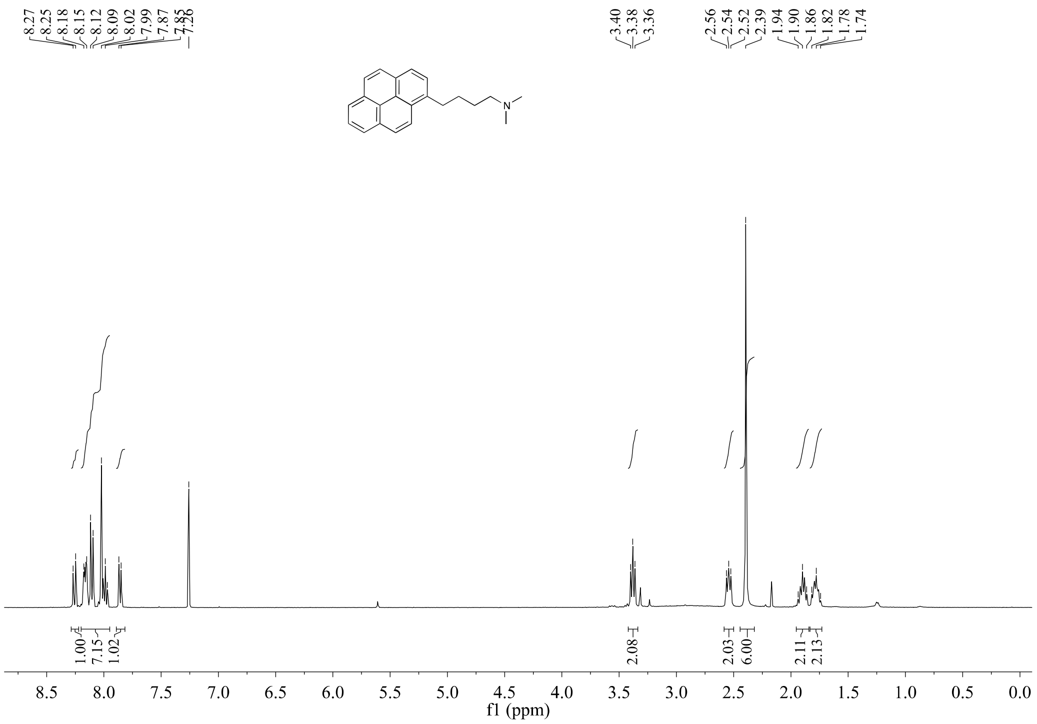

Figure S1: ^{1}H-NMR spectra of **PyNMe2**

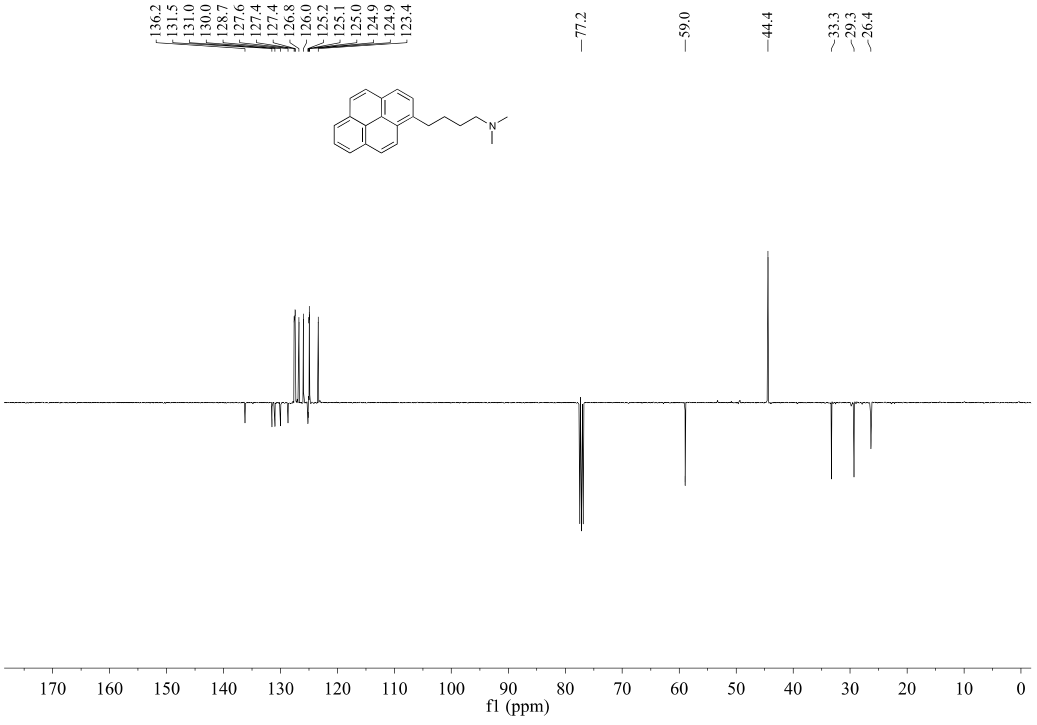

Figure S2: ^{13}C-NMR spectra of **PyNMe2**

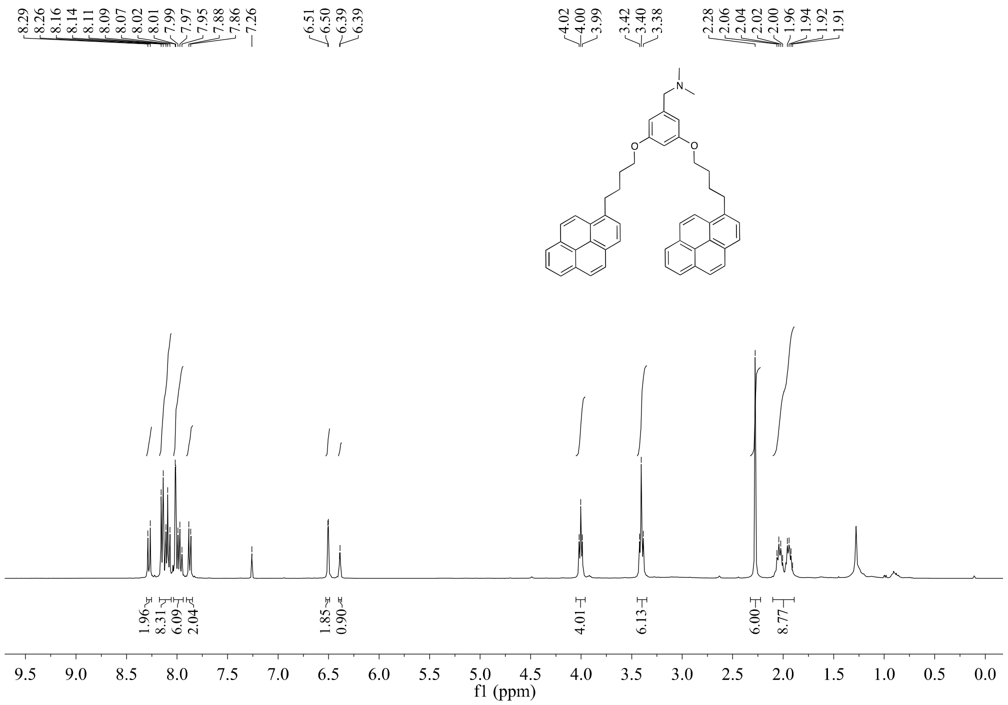

Figure S3: ^{1}H-NMR spectra of **F1NMe2**

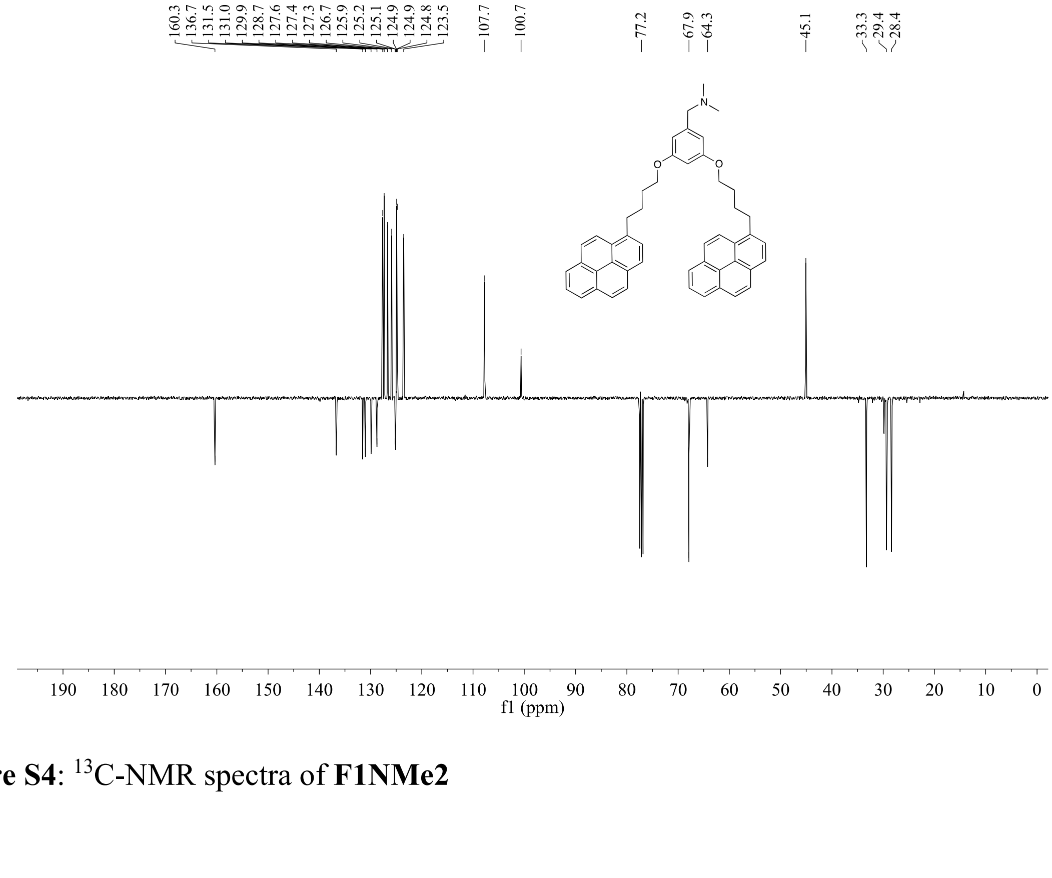

Figure S4: ^{13}C-NMR spectra of **F1NMe2**

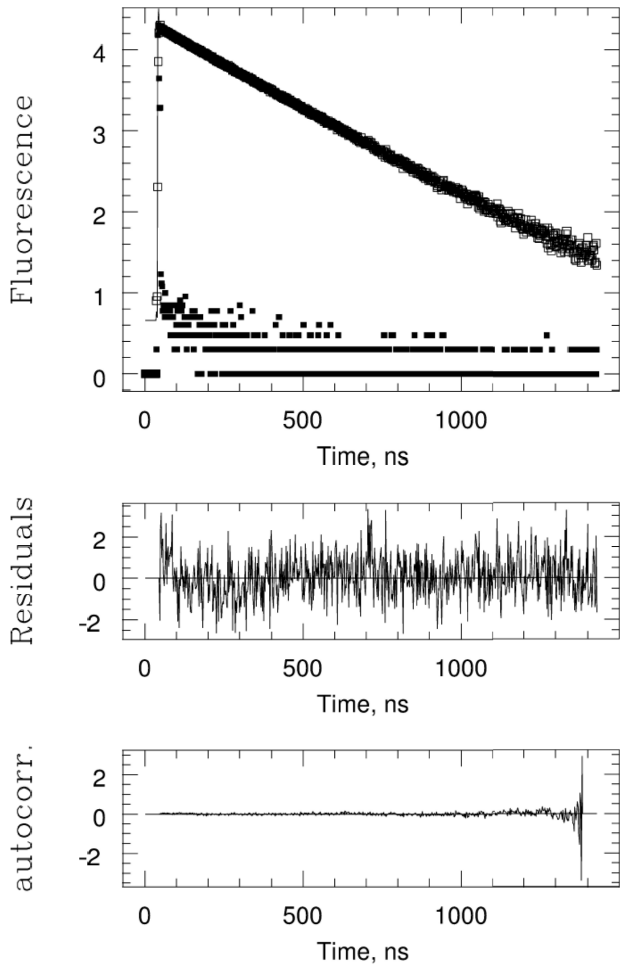

Figure S5. SPC of control compound PyOH

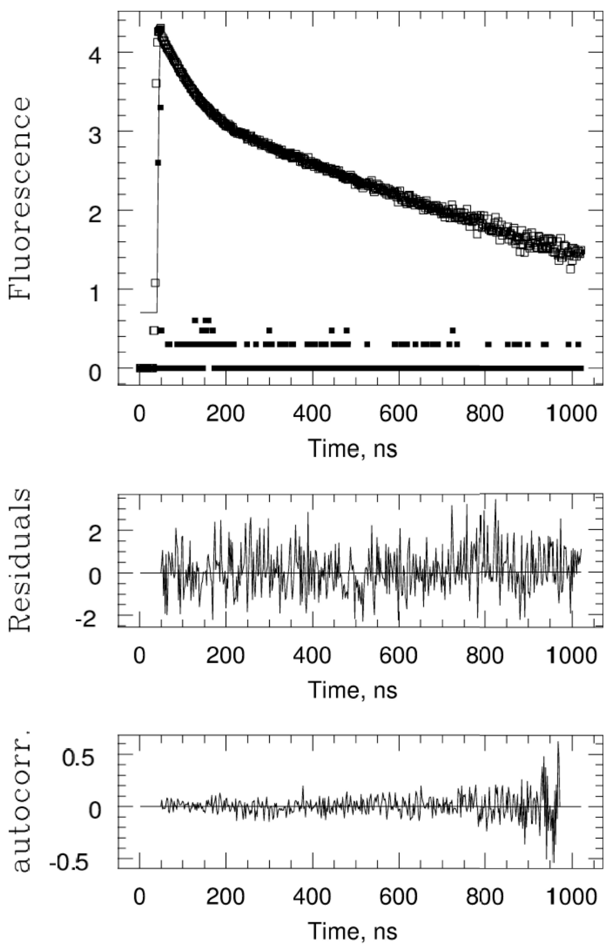

	a_1	τ_1	a_2	τ_2
PyNMe2	0.885	36.06	0.115	209.41

Figure S6. SPC of control compound PyNMe2

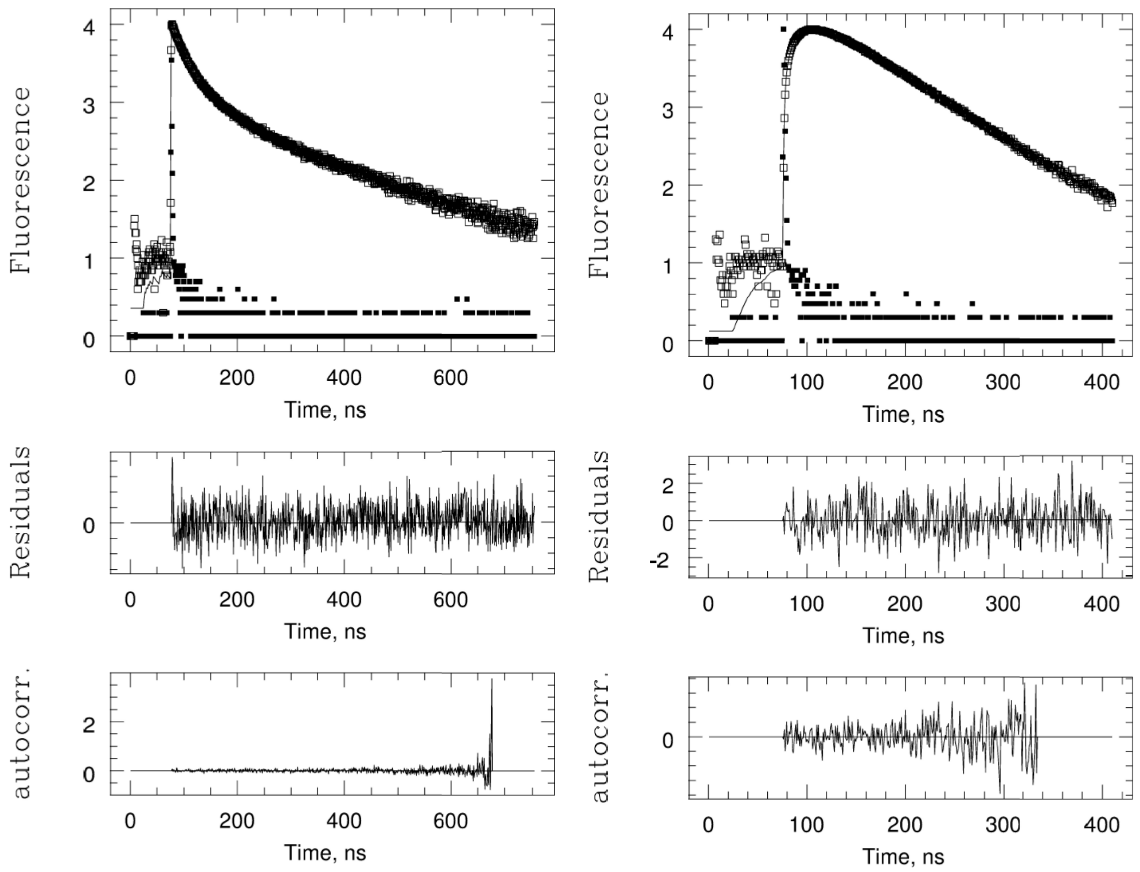

Figure S7. SPC of control compound F1OH. On the right the monomer decay and on the left the excimer decay.

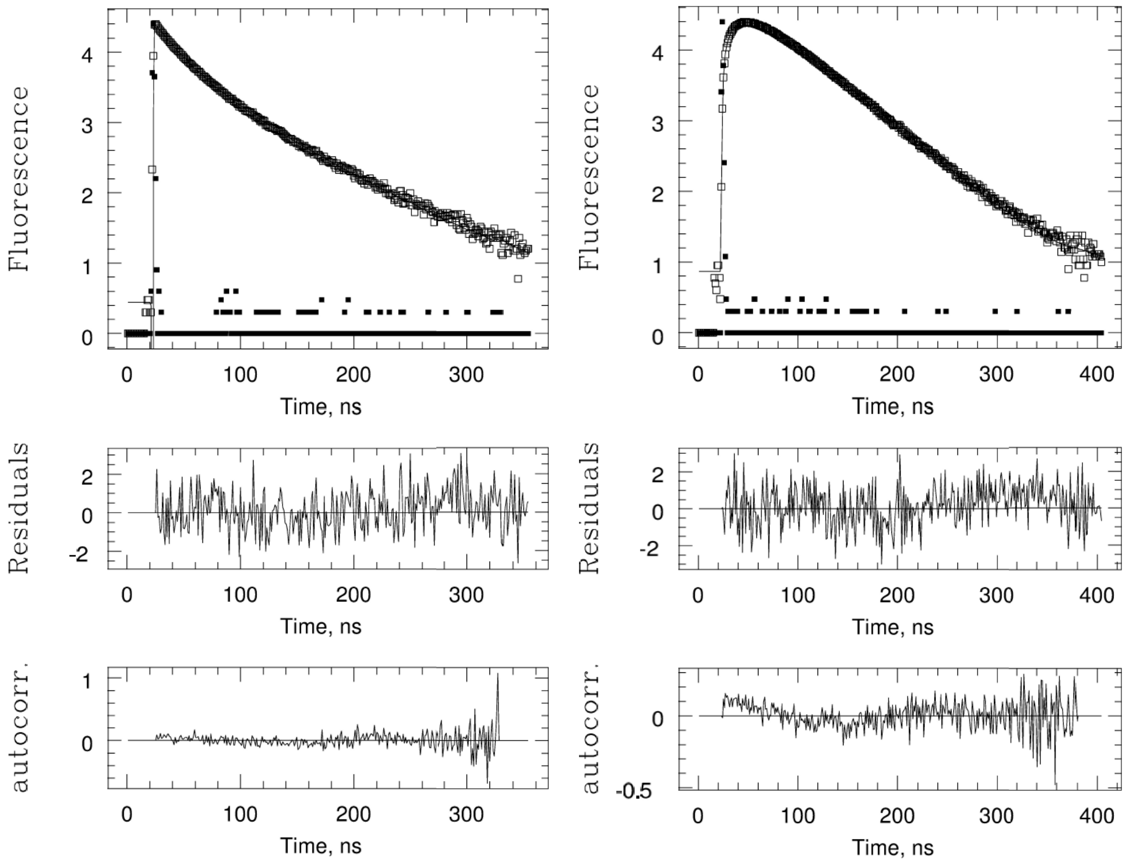

Figure S8. SPC of control compound F1NMe2. On the right the monomer decay and on the left the excimer decay.

THF analysis

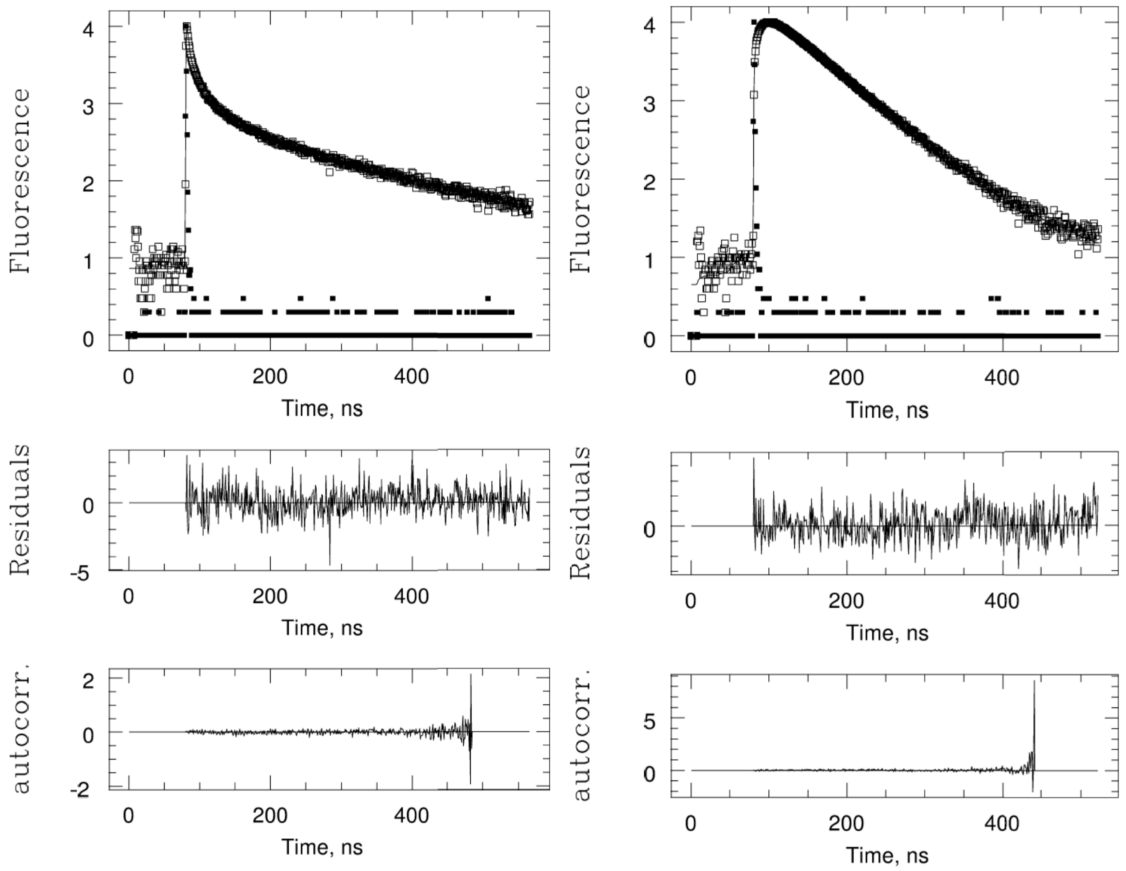

Figure S9. SPC of compound CyPy4 in THF. On the right the monomer decay and on the left the excimer decay.

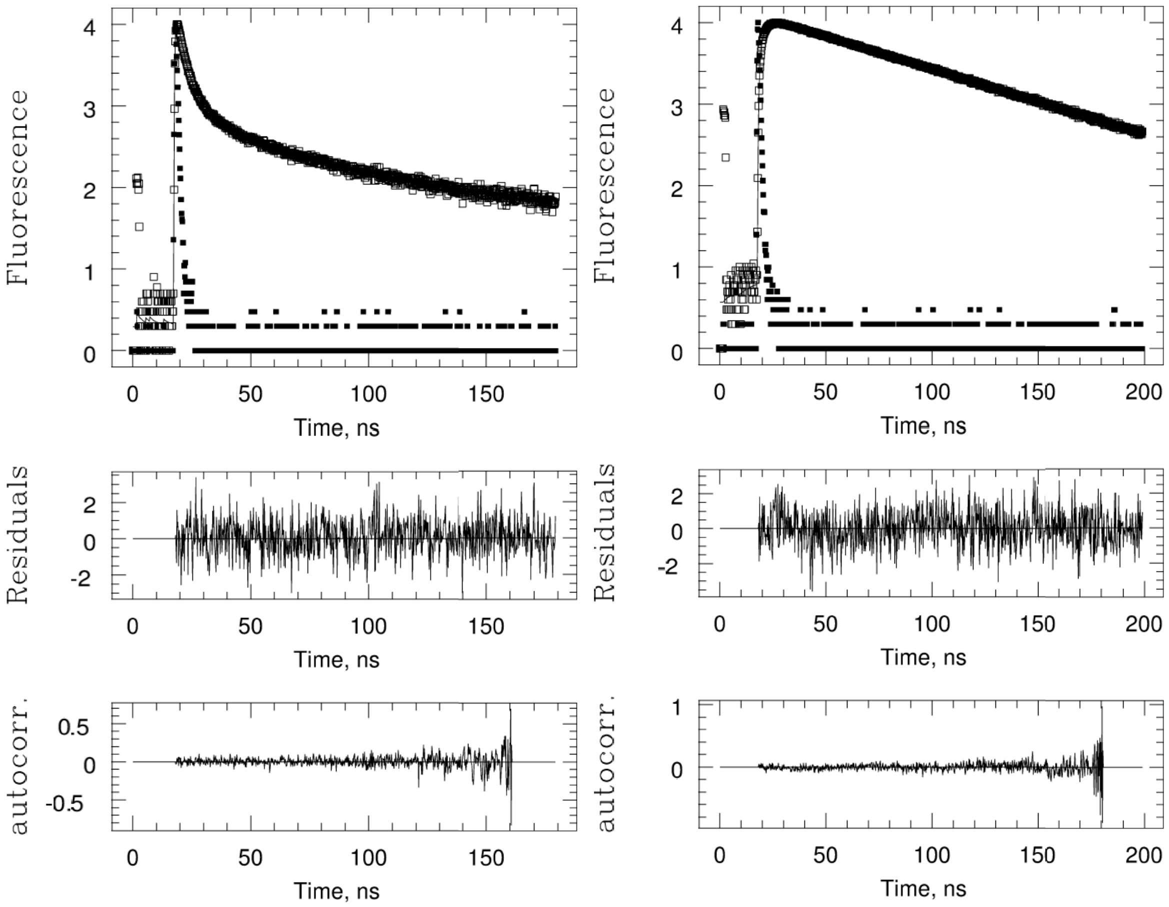

Figure S10. SPC of compound CyPy8 in THF. On the right the monomer decay and on the left the excimer decay.

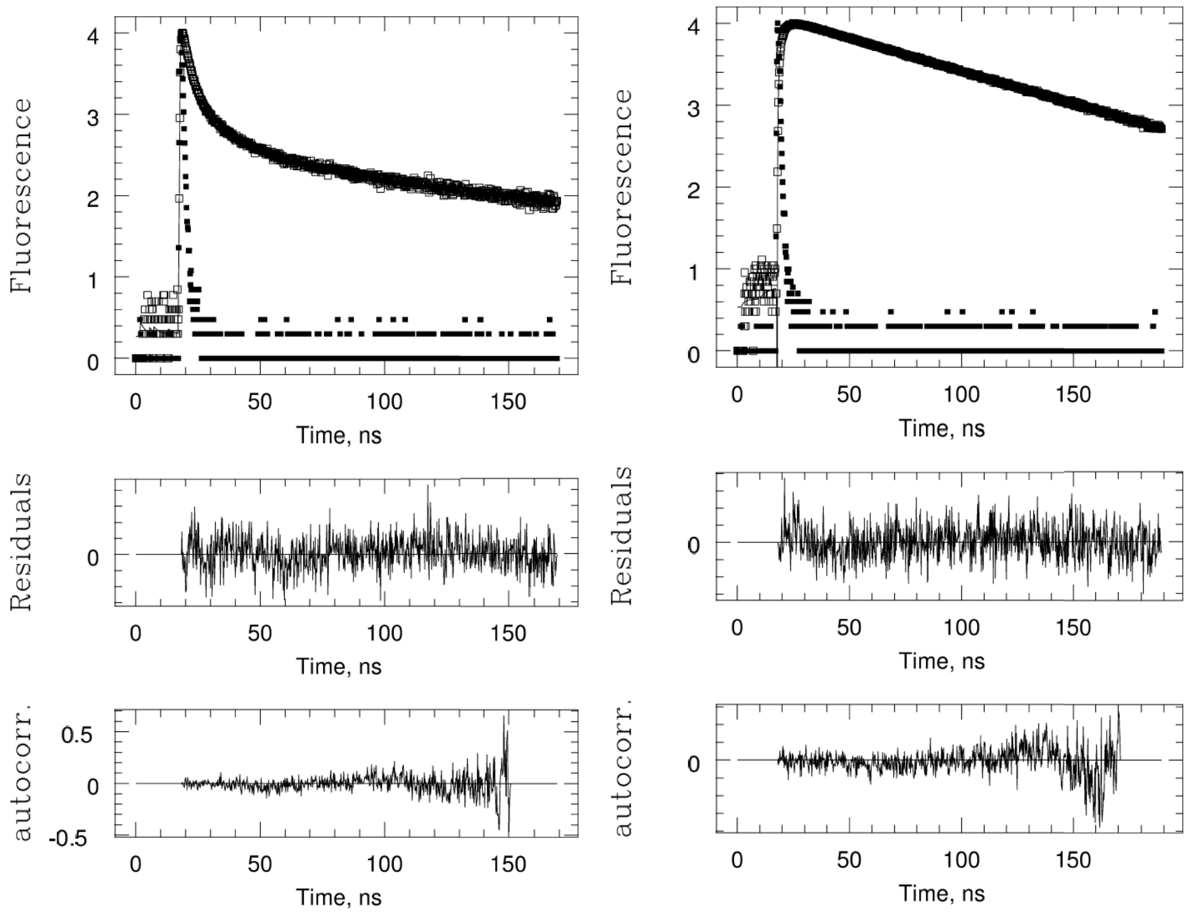

Figure S11. SPC of compound CyPy16 in THF. On the right the monomer decay and on the left the excimer decay.

DMF analysis

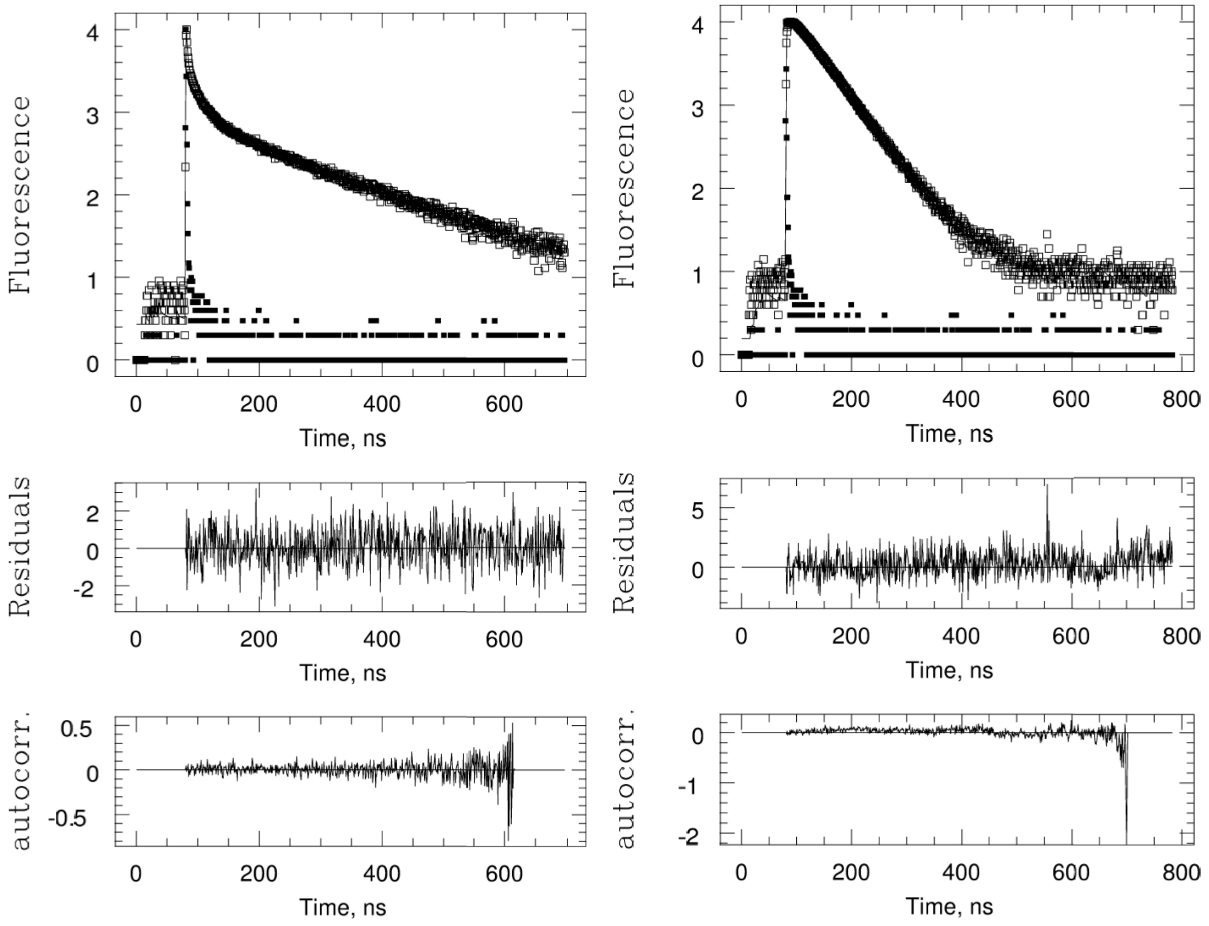

Figure S12. SPC of compound CyPy4 in DMF. On the right the monomer decay and on the left the excimer decay.

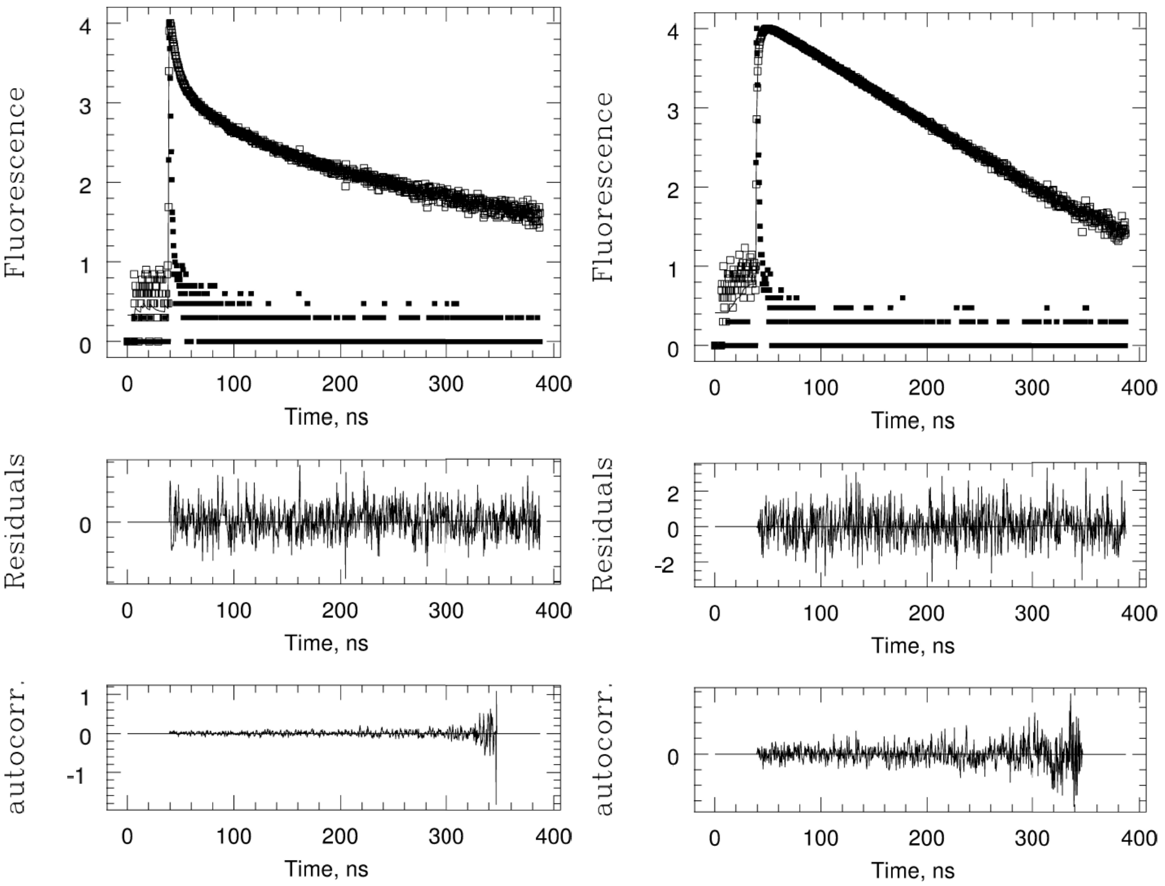

Figure S13. SPC of compound CyPy8 in DMF. On the right the monomer decay and on the left the excimer decay.

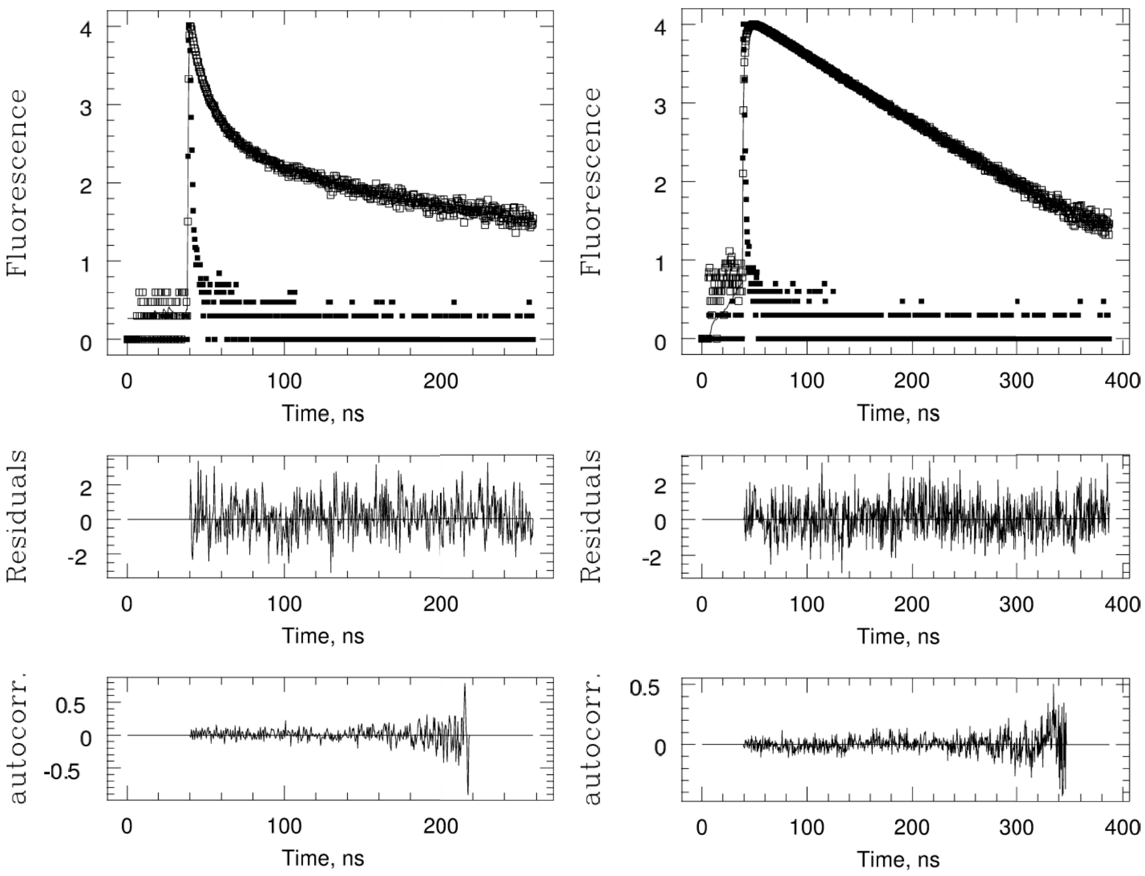

Figure S14. SPC of compound CyPy16 in DMF. On the right the monomer decay and on the left the excimer decay.

DMSO analysis

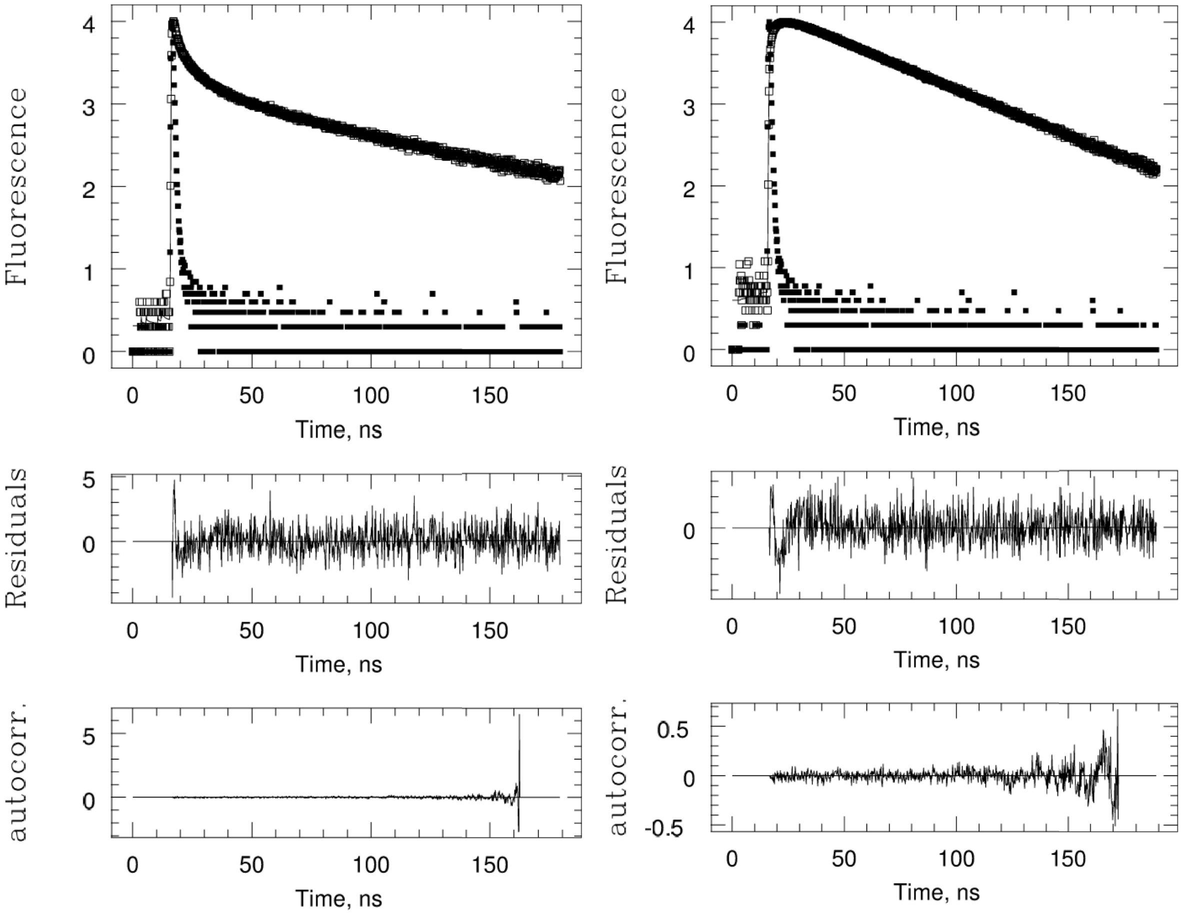

Figure S15. SPC of compound CyPy4 in DMSO. On the right the monomer decay and on the left the excimer decay.

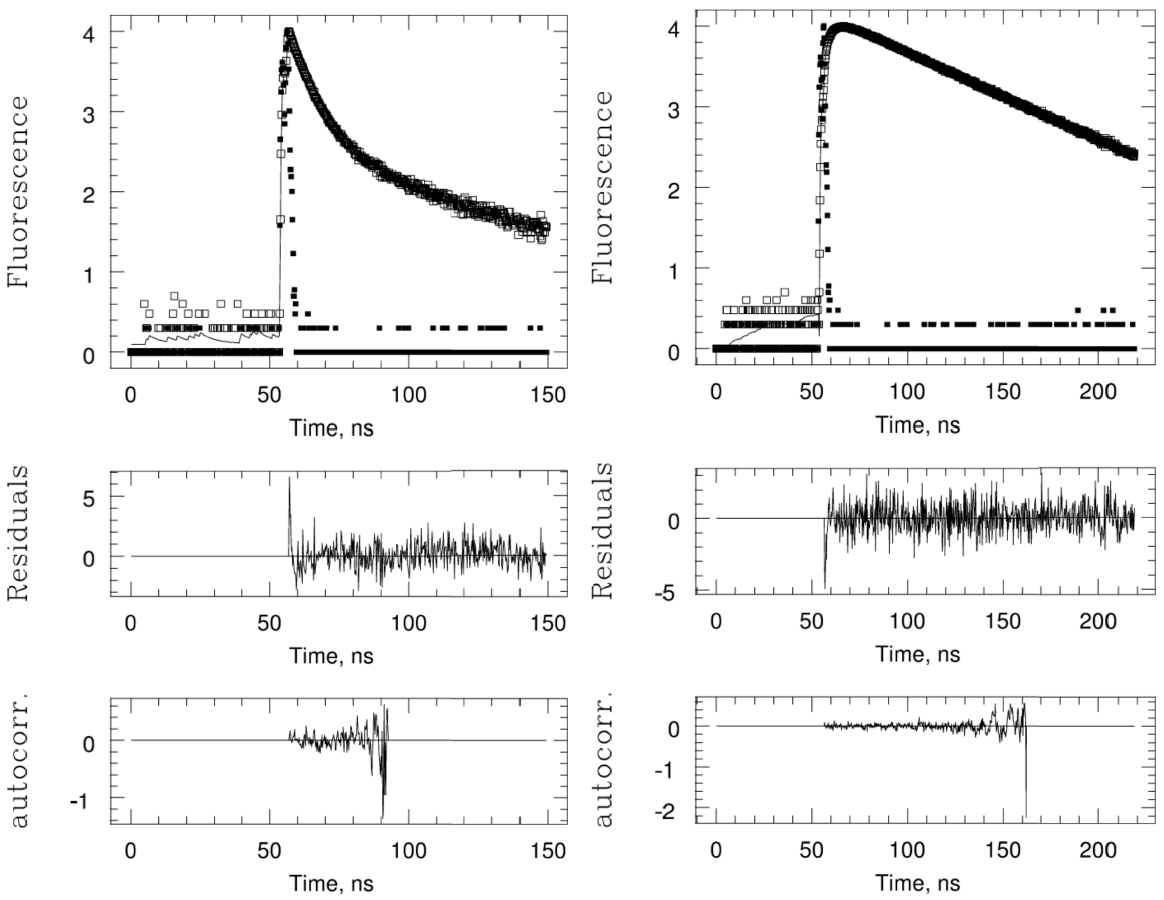

Figure S16. SPC of compound CyPy8 in DMSO. On the right the monomer decay and on the left the excimer decay.

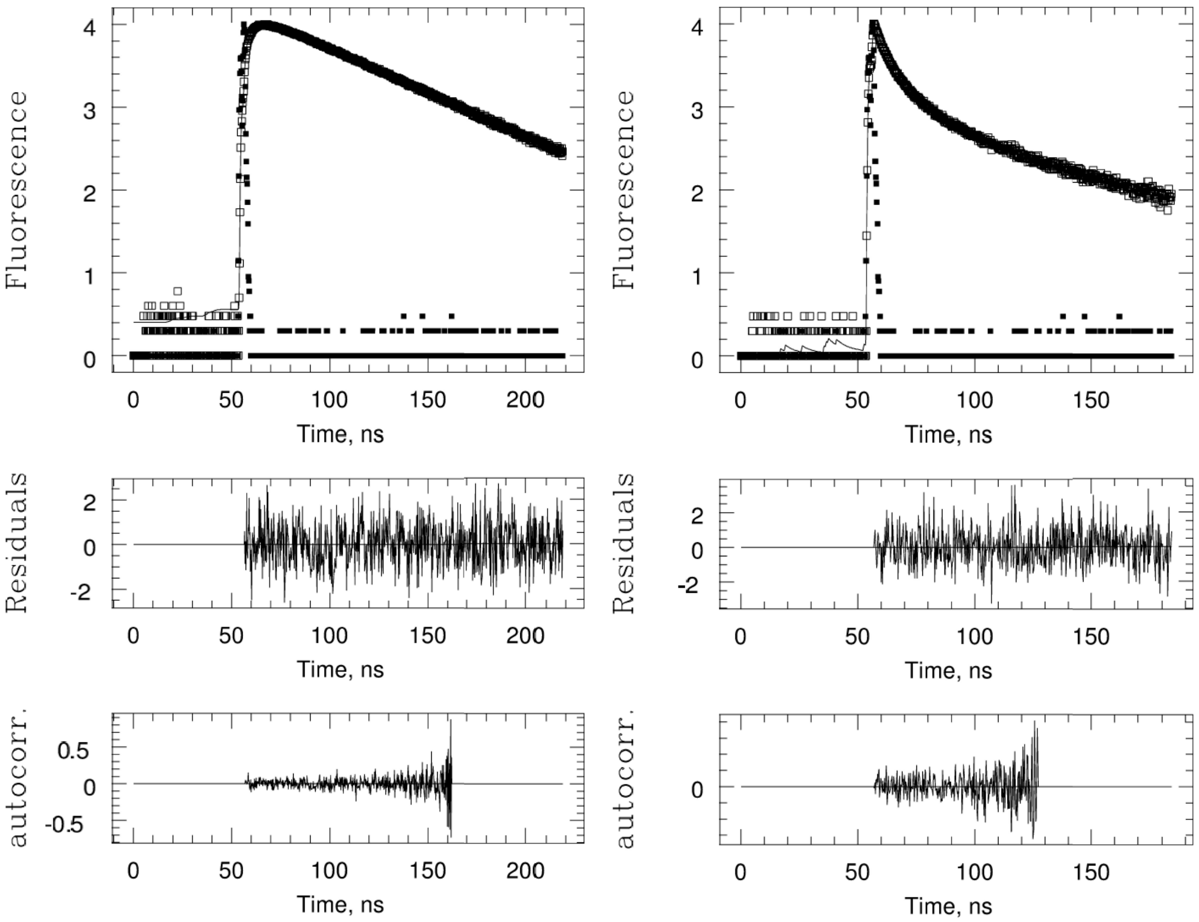

Figure S17. SPC of compound CyPy16 in DMSO. On the right the monomer decay and on the left the excimer decay.

Quenching studies

THF

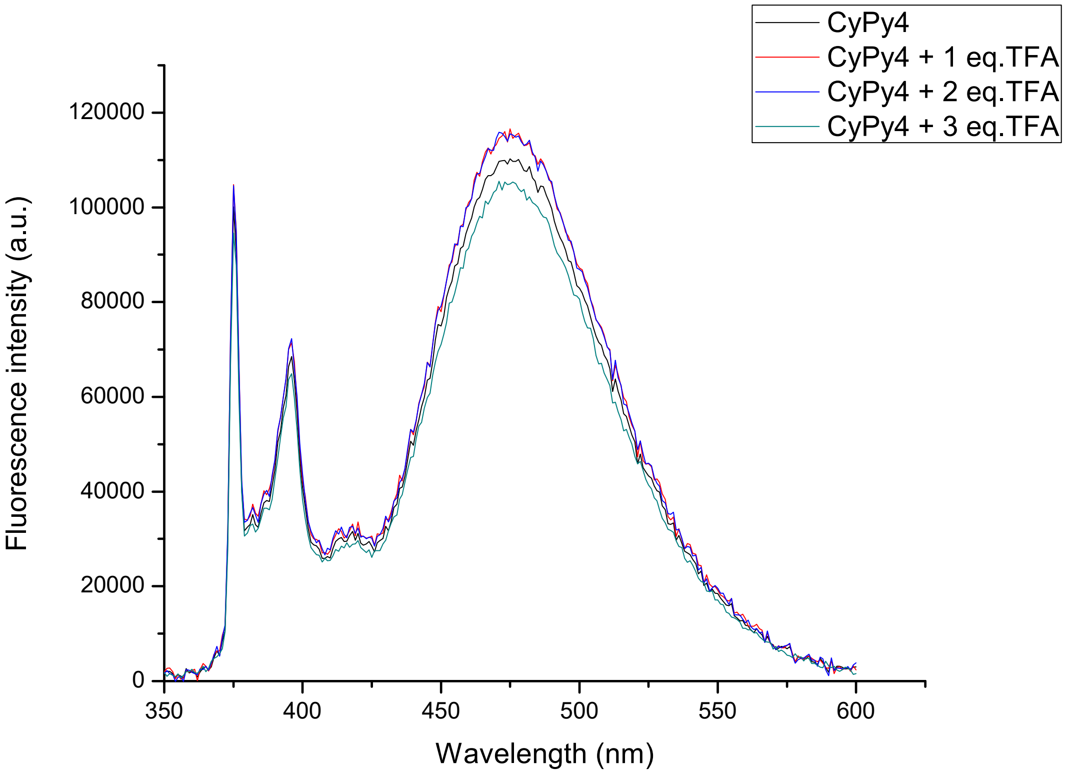

Figure S18. SSF of compound CyPy4 in THF

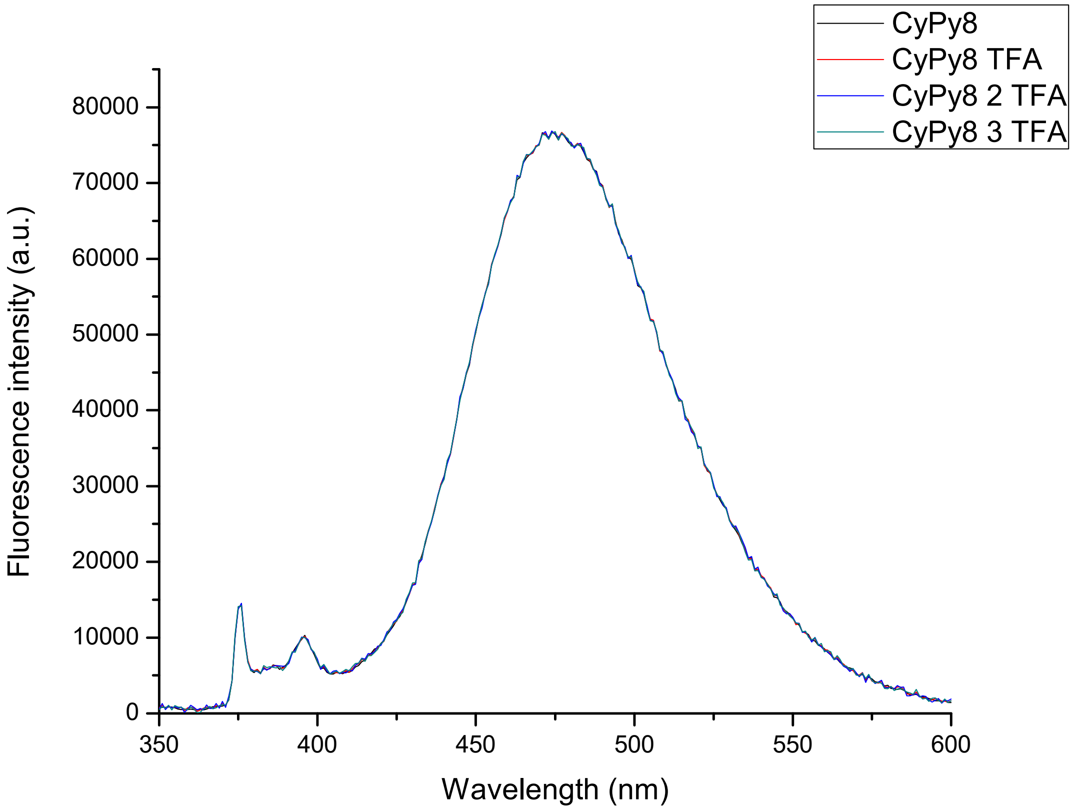

Figure S19. SSF of compound CyPy8 in THF

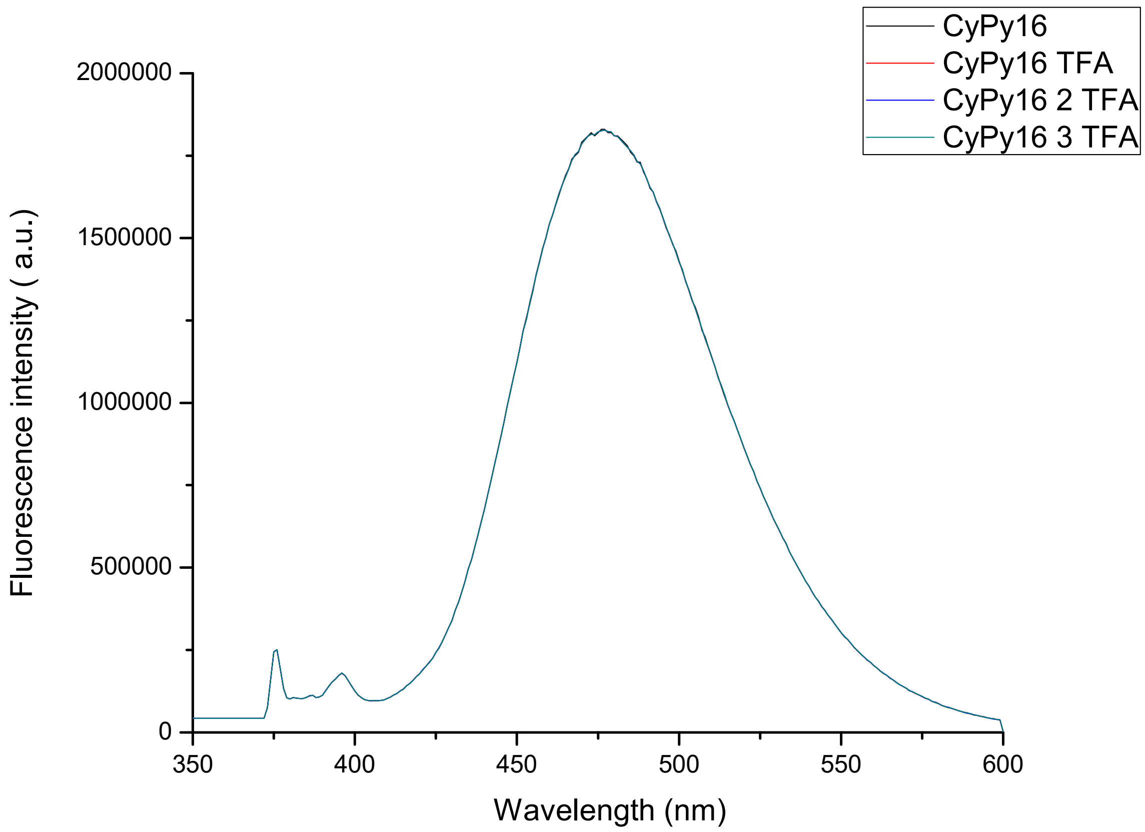

Figure S20. SSF of compound CyPy16 in THF

DMF

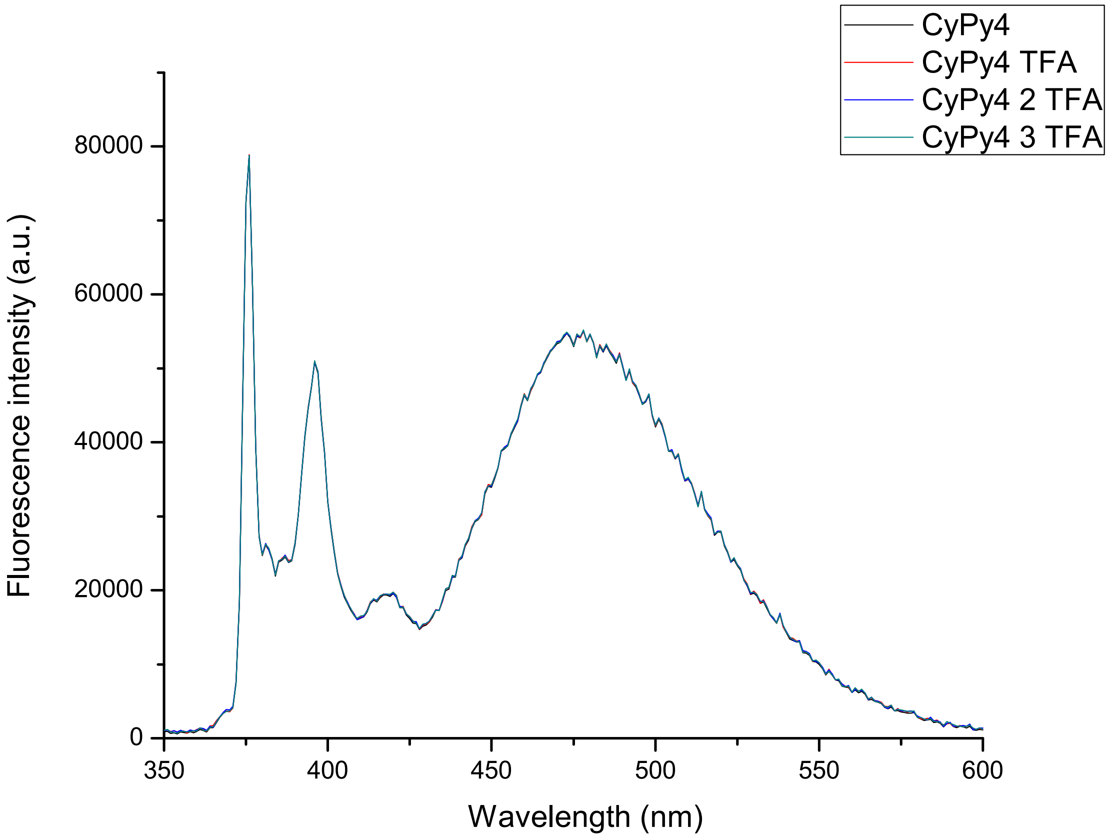

Figure S21. SSF of compound CyPy4 in DMF

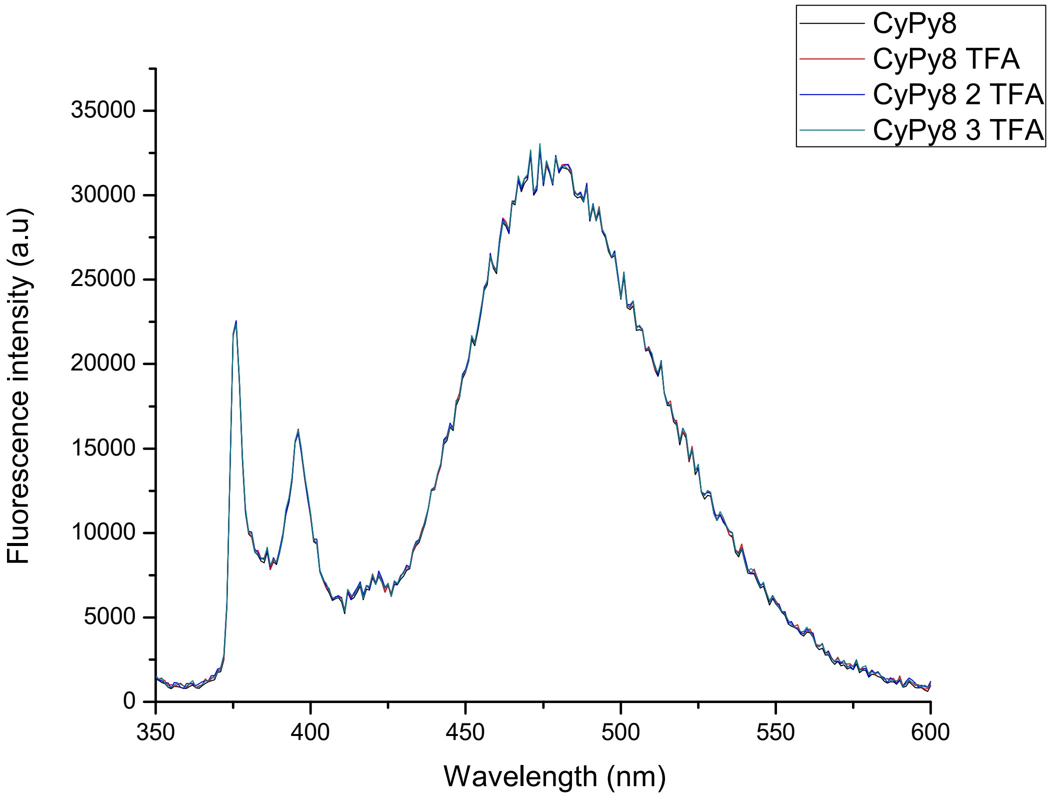

Figure S22. SSF of compound CyPy8 in DMF

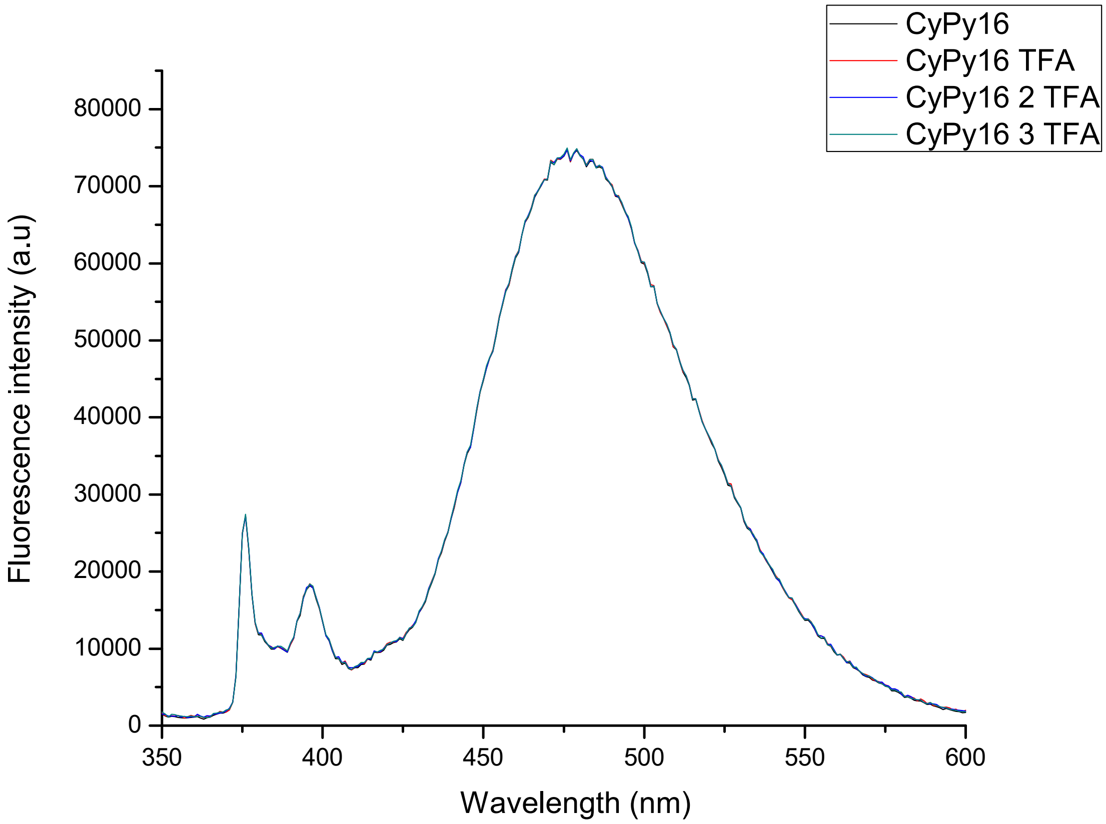

Figure S23. SSF of compound CyPy16 in DMF

DMSO

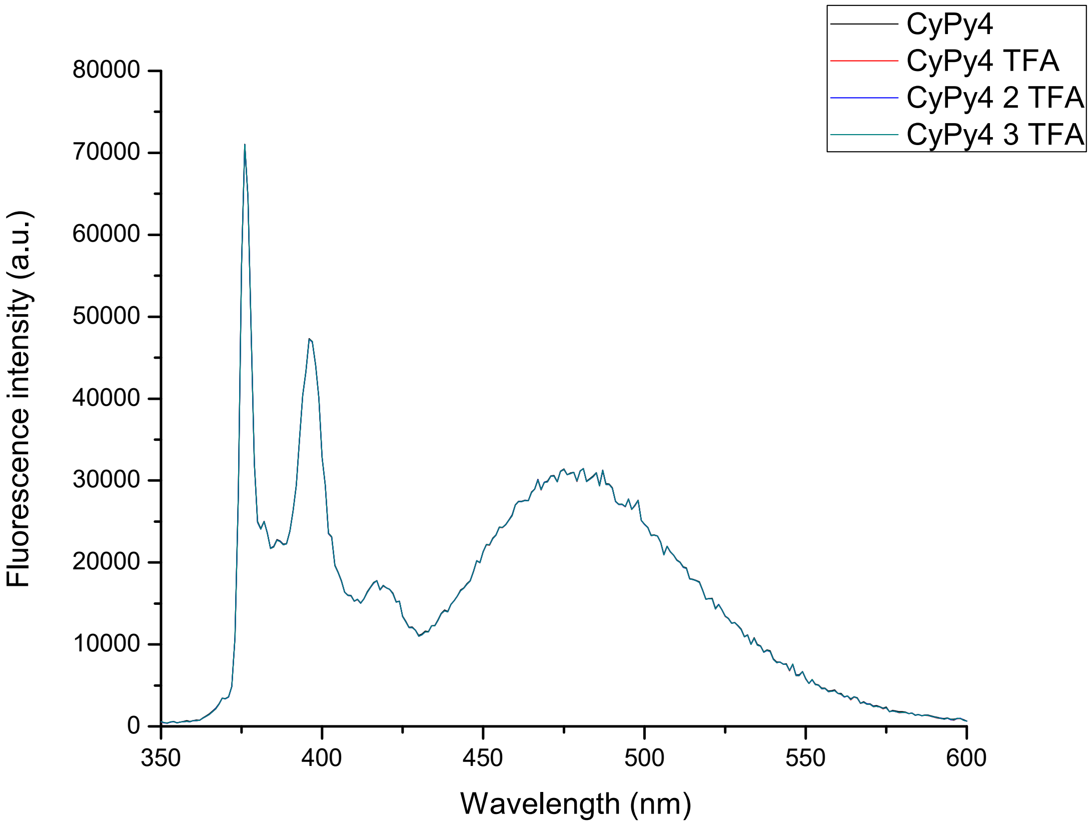

Figure S24. SSF of compound CyPy4 in DMSO

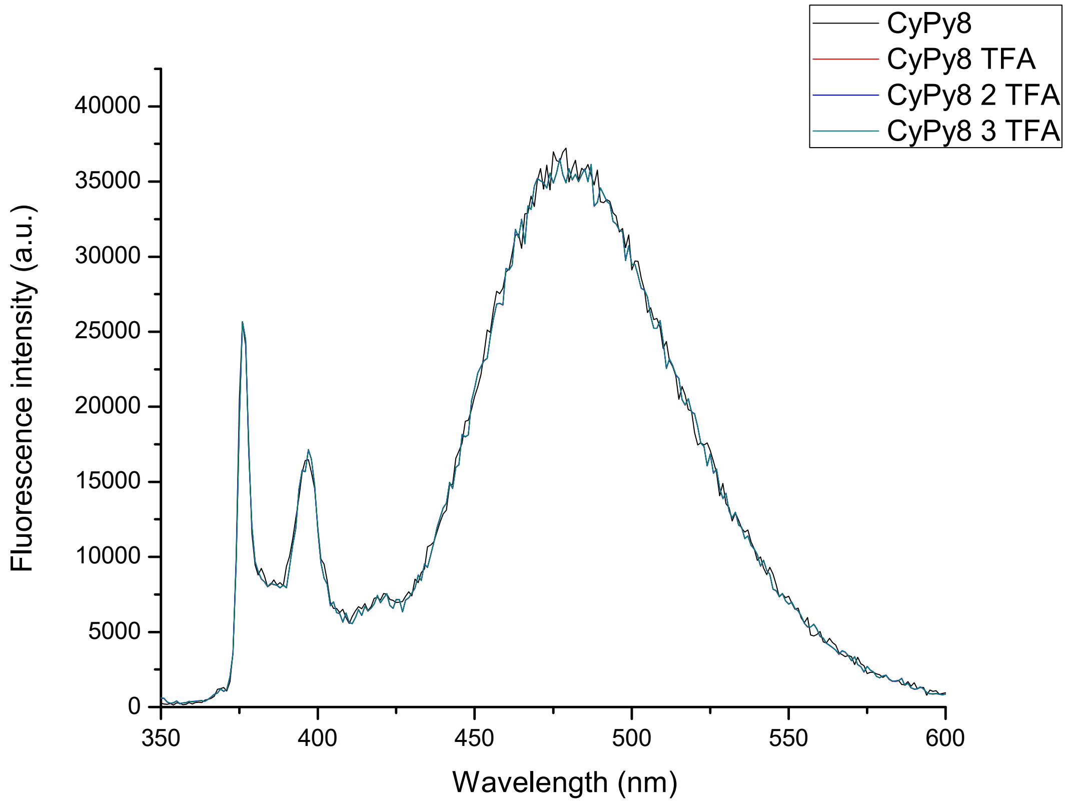

Figure S25. SSF of compound CyPy8 in DMSO

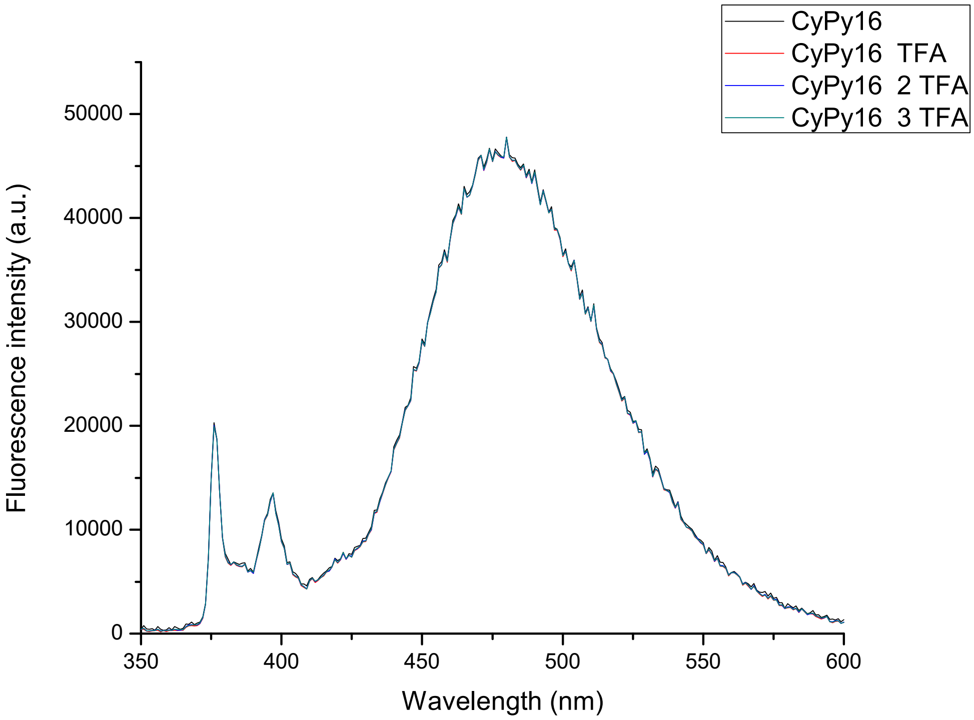

Figure S26. SSF of compound CyPy16 in DMSO

References

[1] G. Zaragoza-Galán, M.A. Fowler, J. Duhamel, R. Rein, N. Solladié, E. Rivera, Langmuir 28 (2012) 11195–11205.

[2] M. Vonlanthen, A. Cevallos-Vallejo, E. Aguilar-Ortíz, A. Ruiu, P. Porcu, E. Rivera, Polymer 99 (2016) 13–20.

[3] A. Cevallos-Vallejo, M. Vonlanthen, P. Porcu, A. Ruiu, E. Rivera, Tetrahedron Lett. 58 (2017) 1319–1323.
